# Designing Governance Boundaries for Hospital Bed Management: Balancing Centralized Pooling and Local Protection

**DOI:** 10.3390/healthcare14131949

**Published:** 2026-07-01

**Authors:** Shao Wang, Feng Xiao, Kin Keung Lai, Yang Xu

**Affiliations:** 1 Competition Training Department, Xi’an Physical Education University, Xi’an 710068, China; 2 International Business School, Shaanxi Normal University, Xi’an 710119, China; 3 Department of Data and Systems Engineering, The University of Hong Kong, Hong Kong; 4 School of Economics Management, Xi’an Technological University, Xi’an 710021, China

**Keywords:** hospital bed management, healthcare management, capacity pooling, centralized bed allocation, local protection, operational resilience, governance boundary

## Abstract

**Background/Objectives:** Hospital-wide bed pooling is widely used in bed-management reform to reduce ward-level mismatch and improve the use of scarce inpatient capacity. However, formally pooled beds are not automatically usable beds. Cross-ward reassignment may require coordination, placement judgment, and timely execution, especially when several wards are under pressure. This study examines hospital bed governance as a healthcare management problem of where to draw the boundary between local protection and centralized pooling. **Methods:** We develop an α-based governance framework that places full decentralization, bounded centralization, and full centralization within a common design space. The parameter α determines how much ward-level capacity is pooled and how much remains locally protected. The analysis first establishes a frictionless pooling benchmark, and then introduces coordination friction, stress-state pooled-allocation imperfection, and the protective value of local capacity. Scenario-based computational experiments examine the mechanism across operating conditions, alternative friction specifications, and multi-ward extensions. **Results:** In the frictionless benchmark, full centralization weakly dominates because it has the broadest feasible allocation set. When pooled allocation remains fully effective, full centralization is not substantively outperformed. Coordination friction can move the raw maximizing boundary inward, but this movement does not necessarily imply a meaningful improvement over full centralization. When reassignment becomes less reliable in high-pressure states, however, an interior governance boundary can become substantively attractive by preserving some locally accessible capacity. **Conclusions:** The findings do not reject centralized pooling. They suggest that bed reform should evaluate both the formal scope of pooling and the operational usability of pooled capacity under pressure.

## 1. Introduction

### 1.1. Hospital Bed Mismatch and Centralized Pooling

Inpatient bed pressure is often treated as a capacity and patient flow problem. In practice, however, the difficulty is not always a shortage of beds in the hospital as a whole. Some wards may have unused beds while others are unable to admit patients in time. Such mismatch can arise from uneven admissions, discharge timing, continuing occupancy, and the organization of beds within clinical units rather than as a fully shared stock. Prior work on patient flow and hospital capacity planning has shown that congestion, delayed admissions, and bed blocking are closely tied to variability and coordination across hospital units, not only to the nominal number of beds available [1,2,3,4]. The issue, then, is not only how many beds a hospital owns. It also concerns how those beds are made operationally available when demand is uncertain and uneven across wards.

A bed listed on the board is not always a bed that can be used for a patient elsewhere. Before reassignment, staff still need to check whether the receiving ward can accept the patient, whether the team on duty has the required capacity, and whether the transfer can be completed before the demand state moves on. The frictionless benchmark sets these checks aside. Under that assumption, an observed bed is accepted and converted into a placement without delay, which gives full centralization the broadest allocation set after demand is realized. The benchmark is useful for defining that upper bound. It should not be read as a close account of daily bed work. On busy days, system visibility can run ahead of authority, staffing, and clinical fit. A bed may sit inside the shared pool while remaining practically unavailable, even as wards remain short and placement decisions wait for clearance.

### 1.2. Pooling Efficiency Versus Local Protection

Under routine conditions, the work needed to make a pooled bed usable is often hidden. A ward may show spare capacity, yet that capacity still has to be released, matched to the patient, accepted by the receiving ward, and absorbed into the ward’s current workload. These checks may be quick. They become more consequential when pressure rises in several units at the same time.

Placement staff then have to compare competing requests, and a bed visible at the hospital level may not be released quickly enough for the shortage already forming. Patient flow studies describe congestion in similar terms. Variability, timing, and coordination across units shape access alongside average demand and nominal capacity [3,4,5,6].

The model represents this gap through the governance boundary and the reassignment work generated after that boundary is set. The parameter α determines how much ward capacity enters the shared pool and how much remains within immediate ward access. When pooled beds are used, Q records the coordination work attached to that use. The stress-state experiments add a further constraint by allowing pooled capacity to become less reliable when several wards are under pressure together.

Local protection has value only in this operational setting. Capacity kept under ward access can be used through familiar staffing arrangements, specialty routines, and local judgments about patient need. Its advantage lies in speed and reachability, before a centrally coordinated reassignment has been identified, approved, and completed.

### 1.3. From Regime Comparison to Governance Boundary Design

In practice, bed governance is rarely a clean split between ward control and a single hospital-wide pool. Many hospitals keep some beds within ward routines for immediate admission, while making other beds visible to a placement office for cross-service reassignment. A binary description of reform can obscure this mixed arrangement. The more relevant design question is how much access should remain close to the ward, and how much capacity should be placed under central coordination before demand is realized.

A possible match still has to become an actual placement. Operations management research makes the same point in broader terms. Pooling and flexibility create value when information, decision rights, and operating routines allow the match to be carried out in practice [7,8,9]. The frictionless case deliberately removes this difficulty by treating every pooled bed as reliably available. When wards are already under pressure, however, reassignment becomes less automatic. A coordinator may see a bed, but authority has to be confirmed, clinical fit checked, placement work completed, and transfer timing arranged. If several wards are competing for the same capacity, these steps may slow down or break down. The practical issue for managers is therefore where to draw the boundary between capacity that wards can use at once and capacity that must move through shared allocation.

### 1.4. Research Questions

The preceding discussion leads to three research questions.

First, when pooled capacity can be reassigned without delay, coordination cost, or loss of clinical fit, does full centralization remain the natural benchmark?

Second, when reassignment becomes costly or less dependable under stress, how is the advantage of full centralization affected?

Third, under what operating conditions can an interior governance boundary become attractive, and does this mechanism remain visible beyond the two-ward canonical case?

These questions define the scope of the paper. We do not assume that bounded centralization is generally superior to full centralization. Instead, we first establish the frictionless pooling benchmark, and then examine how costly or less reliable reassignment, together with protected local access, can shift the preferred governance boundary.

### 1.5. Methodological Approach

We study these questions using a mechanism-driven analytical model that isolates the governance trade-off, complemented by scenario-based computational experiments. The aim is not to calibrate a predictive model for a particular hospital, nor to determine whether decentralization or centralization is always preferable. Instead, the stylized setting allows us to examine when the usual pooling argument holds, and when costly or unreliable reassignment may make a bounded governance design relevant.

We begin with a general multi-ward formulation because the boundary problem is not confined to two units. Ward capacity, demand variability, and cross-ward dependence need to be represented in a common setting before the governance regimes can be compared. For the analytical arguments, however, we deliberately reduce the setting to a two-ward canonical case. This simplification is useful because it shows the central trade-off in the smallest setting where cross-ward pooling can actually arise. It should not be read as a description of how real hospital bed systems are organized.

The analysis first gives pooling its strongest case. Every bed placed in the shared pool is assumed to be reachable, clinically suitable, and reassigned without delay. Under this idealization, full centralization has the widest feasible allocation set and weakly dominates bounded designs. This benchmark is useful precisely because it shows what pooling can achieve before hospital execution enters the problem. After the ideal case has done its work, the analysis relaxes it. Cross-ward reassignment may involve approval, matching, and transfer coordination. A ward may also need capacity it can use without waiting when pressure builds. The question then becomes practical. Can a bed in the shared pool still be made usable within the decision window?

Correspondingly, across the runs, the degree of centralization is varied first. The model then adds coordination work and reliability loss under stress to observe how performance changes. The purpose is to trace the mechanism. Other runs replace the coordination friction measure or expand the ward system. These checks test whether the interior-boundary pattern is observed only in the two-ward example or still appears under nearby modeling choices.

### 1.6. Contributions and Paper Organization

First, the paper reframes hospital bed management as a governance-boundary problem. Much of the existing discussion treats bed-system resilience as a matter of holding additional buffer capacity, protecting reserve beds, or deciding where overflow capacity can be shared. By contrast, the total bed stock is taken as fixed, and the managerial question is how access to that stock should be organized before demand is realized. In this sense, the α-boundary does not create redundant capacity; it determines how much capacity remains immediately accessible to wards and how much is opened to hospital-wide pooling.

Second, the paper develops a multi-ward framework that places full decentralization, bounded centralization, and full centralization on the same governance continuum. This framework is related to, but distinct from, clustered allocation approaches in which the sharing structure is usually specified through predefined service-line groups, overflow links, or specialty clusters. Here, the boundary of sharing is itself the object of design. The two-ward case is used for analytical clarity, and multi-ward computational extensions then examine whether the same mechanism remains visible beyond the simplest setting.

Third, the paper identifies conditions under which bounded centralization can become managerially attractive without dismissing the value of pooling. The analysis establishes a frictionless benchmark in which full centralization weakly dominates, and then examines how coordination friction, stress-state reassignment limits, and protected local access can jointly shift the preferred governance boundary. The same logic also reflects a practical feature of bed-management reform: a bed that is visible to a central team still has to be made available through information, authorization, placement judgment, and ward-level execution.

The remainder of the paper proceeds from literature positioning to model construction, analytical mechanisms, and computational evidence. Section 2 reviews the related literature; Section 3 and Section 4 introduce the governance setting and model formulation; Section 5 and Section 6 present the analytical and computational results; and Section 7 and Section 8 discuss implications and conclude.

## 2. Literature Review and Research Positioning

### 2.1. Hospital Bed Allocation and Inpatient Capacity Management

For the boundary question studied in this paper, the bed-management literature is useful not primarily because it counts beds, but because it shows how difficult it is to make beds operationally available at the right time and in the right place. Admission, discharge, and transfer decisions shape patient flow across emergency departments, intensive care units, and inpatient wards [10]. Broader work on hospital capacity planning extends the same issue to a longer planning horizon, linking bed management to capacity planning, daily occupancy control, and hospitals’ ability to respond to fluctuating demand [11]. Together, these studies provide the operational background for this paper: congestion and delayed admissions arise not only from the number of beds, but also from how capacity is accessed and coordinated across units.

A related stream of this literature has developed tools for managing beds under uncertain demand and occupancy. Data-driven admission control models use patient information and occupancy states to support placement decisions when reusable beds are limited [12]. In simulation studies, bed management is usually set within changing operating states, not treated as a fixed bed count. For short-term decisions, model inputs come on the one hand from real-time system status and length of stay information, and on the other hand from early warning signals [13]. Other studies examine resource balancing under severe pressure. Ring fencing during the COVID-19 pandemic is a typical case [14]. Taken together, these studies partly emphasize how changing occupancy conditions may affect timely response, demand anticipation, and patient flow control.

What remains less explicit in this literature is the governance structure within which allocation and response decisions are made. Much of the literature takes the capacity structure as given: beds are scarce resources to be allocated, forecasted, protected, or scheduled. A prior design choice concerns how much capacity remains under immediate ward-level access and how much is opened to hospital-wide pooling. The same physical bed stock can therefore have different operational consequences under full decentralization, bounded centralization, or full centralization. This is where the present study shifts from bed allocation alone to the governance boundary between local protection and centralized pooling.

This boundary is not only a modeling distinction; it is also visible in how hospitals manage beds in practice. Central teams, bed boards, and patient flow systems may improve visibility, but they do not by themselves resolve the operational questions of whether a patient can be placed in a particular ward, whether the receiving team has the required capability, and whether the transfer can be completed in time. The next subsection therefore turns to empirical and implementation evidence on centralized bed management before returning to pooling theory.

### 2.2. Empirical Evidence and Practical Applications of Centralized Bed Management

Studies of hospital command centers and patient flow coordination suggest that centralization is usually built through operational arrangements rather than through a bed-assignment rule alone. In the command center design described by Kane et al., real-time displays, predictive analytics, standard work, and rules-based protocols are combined as part of a systems-engineering approach to hospital flow [15]. Qualitative evidence from leading healthcare providers points in a similar direction, emphasizing central coordination, digital support, transfer structures, and discharge planning as practical supports for hospital-wide flow [16]. For this study, these findings give “centralized bed allocation” a more concrete operational meaning: central counting may improve visibility, but beds can be used across units only when information, authority, and coordination routines are organized to support that use.

In daily hospital operations, counts of available and occupied beds are often the easiest indicators to observe. What can be missed is that a patient placed in an available bed may still be outside the care setting and team support best suited to the case. Off-service placement can ease an immediate capacity shortage, but its clinical fit is not as visible as the bed number itself. A poor match may also carry costs, since Song et al. associate this form of hospital capacity pooling with longer remaining length of stay and higher readmission risk [17]. A similar gap appears in timing. Braaksma et al. show that early bed assignment may mark a bed as allocated before the patient is ready to move [18]. These findings do not reject pooling. They make the pooling claim more conditional. In bed management, bed numbers should not be the only consideration. Ward context, timing, and workable clinical arrangements also matter for decision makers.

Command center evidence points in the same direction. Central coordination may improve particular handoffs in patient flow, although current evaluations do not show that a command center automatically improves flow, data quality, or safety. Mebrahtu et al. report mixed NHS evidence, with some transition times improving and no consistent overall gain in patient flow indicators or data quality [19]. Another interrupted time-series study found limited evidence of patient-safety improvement after implementation [20]. Johnson et al. also report data-quality problems and tension between central coordination and local departmental practice in an artificial-intelligence command center study [21]. Centralization in practice depends on the hospital’s ability to build reliable coordinating routines around the formal structure.

Taken together, these studies suggest that pool size is only part of the design problem. A larger common pool would be the obvious target if centralized bed management were only a question of how many beds to share. Hospital operations make the choice harder. A shared bed must be seen in the system, cleared under placement rules, accepted by a suitable ward, and worked into transfer and care routines that can safely handle the patient. The parameter α captures where this access boundary is drawn. It shows how much capacity is opened to central coordination, but the value of that capacity depends on whether the hospital can turn it into usable placements under its own operating conditions.

### 2.3. Resource Pooling and Centralization

When execution costs are set aside, pooling remains a strong starting point. Demand shocks are uneven across wards, and a common service base can absorb some mismatch that dedicated capacity would leave unresolved. Queueing studies give the formal version of this argument by comparing pooled and dedicated service structures. The same literature also cautions against treating the gain as automatic, since heterogeneity and operating conditions can change its size [22]. Studies of differentiated services add another qualification. Even after resources are pooled, allocation rules still shape who receives access and where the burden of sharing falls [23]. In this paper, these results are used for a limited purpose. They establish the clean case in which full centralization is the natural benchmark, but they do not close the hospital design question.

Hospital placement adds a further step between a nominal match and an actual admission or transfer. A bed may be listed, but the patient must be ready, the placement decision cleared, and the receiving ward able to take the case in time. Evidence on geographic virtual pooling speaks to this point. Wider matching can shorten waiting, yet it may also require movement across organizational or spatial boundaries [24]. When transfer timing, clinical fit, or ward-level approval makes reassignment less reliable, the issue is no longer just whether pooling helps in principle. It is how far pooling should extend before some capacity is better kept closer to local use.

### 2.4. Costly Coordination and Organizational Boundaries

A shared bed does not relieve pressure until the placement work has been done. Bed managers first have to find capacity that is usable in practice, confirm that the receiving ward can take the case, apply the relevant placement authority, and work the move into existing ward routines. On quieter days, these tasks may disappear into ordinary bed management. When several units are asking for capacity at the same time, they become part of the allocation constraint. The model locates coordination cost at this point in the process. The cost is attached to cross-ward reassignment work, rather than to central pooling as a formal arrangement.

Evidence from operations and organization studies supports this placement of the cost. Queue configuration can affect provider behavior because staff respond to queue ownership and to visible queue length. In healthcare delivery experiments, dedicated queues have sometimes processed patients faster than pooled queues without reducing quality when these behavioral channels are active [25]. Centralization studies point to a similar condition. More centralized structures improve adaptation only when communication patterns can carry the necessary information, including feedback from local units [26]. For bed governance, the implication is fairly direct. The burden is not the existence of a shared pool itself. It is the work required to turn a visible bed into an approved and timely reassignment. The model therefore measures coordination cost through the amount of reassignment performed, instead of applying a fixed penalty to the governance regime.

### 2.5. Operational Resilience and Local Buffers

Local reserve is hardest to justify when pressure is low, or when ward-level shocks do not arrive together. In those conditions, a central pool can look more efficient. Beds held near one ward can be shifted toward another ward with a shortage, improving the use of capacity across the hospital. The argument changes when several wards are short at the same time. A ward with a small accessible reserve may be able to admit or hold patients through its own routines while the central team is still locating a bed, confirming that it can be used, and arranging the reassignment. What matters is not simply that extra capacity exists. The point is that the organization retains a usable option after disruption has already begun. This links the model to the resilience literature, where slack is valuable because it helps organizations absorb pressure, preserve options, and recover from stress [27].

Hospital capacity studies place the same idea in a more concrete setting. Work on ICU infrastructure treats slack as one element of resilience management after the pandemic stress test [28]. Surge preparedness reviews describe emergency capacity as a bundle of staff, supplies, space, beds, systems, and the ability to expand or protect usable capacity as conditions worsen [29]. The meaning of local protection in this paper is narrower. It refers to capacity that remains reachable by the ward when shared reassignment slows or becomes unreliable. Full pooling may improve average matching, yet it can leave a ward with fewer local options when pressures become synchronized. Recent calls for models linking surge, demand, coordination, and system response are therefore relevant to this design choice [30]. Local protection is used here to capture the idea that exposure to severe-shortage states depends partly on where usable capacity is held.

### 2.6. Research Gap and Positioning

Off-service placement shows what pooling means once the decision is made inside a hospital rather than in an abstract capacity model. A patient can be moved to a ward with available space, and the immediate bed shortage may ease. At the same time, the patient is now cared for by a different team, under different ward routines, with extra coordination around handover, monitoring, and discharge planning. Empirical studies associate this form of pooling with longer remaining length of stay and higher readmission risk [17]. Related evidence also finds spillover effects for patients who stay on the original service, which indicates that the work consequences extend beyond the patients who are physically reassigned [31]. For this reason, pooling in hospitals should be read as a change in the organization of care, not only as a way to offset uneven demand.

Clustered overflow research has already developed intermediate designs between strict dedication and full pooling. These designs allow overflow capacity to be shared within specialty subsets [32]. Later studies optimize department clustering, dedicated bed allocation, and shared overflow beds under multiple operating costs [33]. Recent work on inpatient service reconfiguration also finds clustered overflow structures attractive under bed pressure [34]. In these models, however, the sharing structure is usually chosen in advance. Departments are assigned to clusters, overflow routes are specified, and dedicated or shared capacity is placed inside that architecture. Redundant bed and buffer capacity models address a neighboring question by deciding how much slack or backup capacity to hold against demand uncertainty.

The framework built around α takes a different design object. It fixes the total bed stock and lets α determine the split between ward access and hospital-wide coordination. As a result, the same model can examine the frictionless pooling benchmark, realized coordination burden, stress-state limits on pooled allocation, the value of local protection, and robustness in multi-ward settings. This paper asks how far centralization should extend when pooled capacity is valuable, its use requires coordination, and local access may help during pressure states. Section 3 formalizes this setting through the bed stock, ward demand, and the centralization boundary.

## 3. Problem Setting and Governance Regimes

### 3.1. Hospital Bed Governance in an n-Ward System

We consider a hospital bed-management setting with n inpatient wards, indexed by i∈I={1,…,n}. Ward i has nominal bed capacity $B_{i}$, and the hospital has a fixed total bed stock

$$B=\sum_{i=1}^{n}\;B_{i}.$$

The focus is not short-term bed expansion, capacity investment, or the creation of redundant beds. Accordingly, we take the hospital bed stock as fixed and examine how access to that stock is governed across wards. The central question is therefore how much capacity should remain under immediate ward-level access and how much should be made available for hospital-wide coordination.

During a planning period, ward i faces random bed demand $D_{i}$. We use $D_{i}$ to denote net bed demand, rather than new arrivals alone. This demand reflects admission pressure, continuing occupancy, and expected bed releases during the planning period. Patient-level length-of-stay (LOS) dynamics are therefore not modeled explicitly. LOS-related turnover is instead absorbed into the distribution of $D_{i}$. This treatment does not imply that LOS or discharge processes are unimportant. It allows the analysis to focus on how a realized net demand state is handled across ward boundaries under different governance arrangements. In this formulation, $D_{i}$ is best read as the net pressure placed on ward i’s bed capacity during the planning period. It is not intended to be a simple end-of-day census, although it may be summarized from data observed over a day or a shift. For empirical implementation, such a variable could be constructed from occupied beds at the beginning of the period, expected or realized admission requests, expected discharges, and transfer-related inflows and outflows. Depending on the available data, $D_{i}$ can therefore be implemented as an admission-equivalent net demand or as a synthetic net-flow variable. This interpretation keeps LOS and discharge dynamics in the background as sources of the realized demand state, while allowing the model to focus on how that state is absorbed through local capacity and centrally coordinated pooled capacity. Let

$$D=(D_{1},\ldots ,D_{n})$$

be the ward-level demand vector. We write

(1) $$D∼F\left(\mu ,\Sigma \right),$$

where $\mu =(\mu_{1},\ldots ,\mu_{n})$ is the mean demand vector and Σ is the covariance matrix. The covariance structure allows demand shocks to be correlated across wards. When ward demands are weakly correlated, spare capacity in one ward is more likely to offset pressure in another. When demands move together, several wards may compete for capacity at the same time, weakening the diversification benefit from pooling.

We describe the governance structure through a centralization degree α∈[0,1]. In managerial terms, α is a planning-level boundary between immediate ward-level access and hospital-wide coordination. When α is low, most capacity remains locally accessible to each ward. When α is high, more capacity is opened to hospital-wide reassignment. Intermediate values represent designs that combine local protection with central pooling. Formally, α represents the share of each ward’s nominal capacity placed in a hospital-wide shared pool. Given α, ward i retains a protected local reserve $r_{i}(\alpha )$, and the hospital-wide shared pool has capacity S(α)

(2) $$r_{i}\left(\alpha \right)=\left(1-\alpha \right)B_{i},\;\;\;\;S(\alpha )=\alpha B.$$

A larger α expands the shared pool but reduces protected ward-level capacity. Thus, α represents the governance boundary between local protection and centralized pooling. This *n*-ward formulation provides the general hospital setting for the paper. Later analytical sections use a two-ward canonical case to make the governance mechanism transparent, while the computational experiments return to multi-ward settings.

Table 1 summarizes the notation and model primitives for the governance framework. The objects especially relevant to the subsequent discussion are the centralization degree α, the protected local reserve $r_{i}\left(\alpha \right)$, the shared-pool capacity $S\left(\alpha \right)$, the realized allocation $x_{i}\left(D,\alpha \right)$, and the performance measure $\Pi \left(\alpha \right)$.

### 3.2. Decision Timing and Information Structure

The decision sequence separates governance design from operational response. Before ward-level demand is realized, the hospital sets the centralization degree α ∈ [0,1]. This choice fixes the governance boundary for the planning period and determines how much capacity remains under immediate ward-level access and how much is opened to hospital-wide coordination.

After demand is realized, the hospital observes the ward-level demand vector

D=(D1,…,Dn)

and chooses an effective capacity allocation within the limits set by the chosen governance boundary α. We denote the resulting allocation by

x(D,α)=(x1(D,α),…,xn(D,α)),

where xi(D,α) is the effective bed capacity available to ward i after the demand state is observed. The local reserve remains available through immediate ward-level access, while capacity in the shared pool must be assigned through the central coordination process.

This timing sequence keeps governance design separate from operational response. The parameter α determines what allocations are feasible once demand is observed; it does not prescribe a fixed allocation for every demand realization. In this sense, α defines the scope of feasible coordination rather than a real-time dispatch rule. After the allocation x(D, α) is chosen, ward-level outcomes such as admitted demand, idle capacity, and unmet demand are determined. Coordination burden and tail-risk exposure are also evaluated at this stage. Section 4 formalizes the feasible allocation set, the outcome definitions, and the performance measures used in the analysis.

### 3.3. Governance Regimes Under the α-Framework

**Figure 1:**
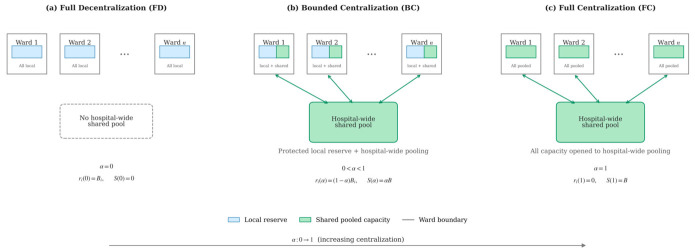
Hospital bed governance under (**a**) full decentralization, (**b**) bounded centralization, and (**c**) full centralization.

The centralization degree α places the three bed-governance arrangements in a common framework. As Figure 1 shows, full decentralization, bounded centralization, and full centralization are not treated as unrelated regimes. They occupy different positions on the same continuum between immediate ward-level access and hospital-wide pooling.

When α=0, all nominal bed capacity remains under immediate ward-level access. When α=1, the entire bed stock is opened to hospital-wide pooling. Values between these two endpoints describe bounded centralization, where each ward retains part of its nominal capacity as a protected local reserve and the remaining capacity is made available for central coordination.

When α=0, the system corresponds to full decentralization (FD). By Equation (2), each ward retains its full nominal capacity, $r_{i}(0)=B_{i}$, and no hospital-wide shared pool is formed, S(0)=0. FD gives wards the strongest immediate local access, but it does not allow hospital-wide rebalancing after demand is realized.

When 0<α<1, the system corresponds to bounded centralization (BC). By Equation (2), each ward retains a positive protected reserve, $r_{i}(\alpha )=(1-\alpha )B_{i}>0$, while the shared pool also has positive capacity, S(α)=αB>0. BC therefore combines central coordination with protected local access. It keeps hospital-wide pooling in place, but it does not place all capacity under the shared pool.

When α=1, the system corresponds to full centralization (FC). By Equation (2), no ward-specific reserve is protected, $r_{i}(1)=0$, and the full bed stock is placed in the shared pool, S(1)=B. FC provides the widest scope for hospital-wide pooling and serves as the natural benchmark when sharing is frictionless.

With this nesting, FD, BC, and FC are no longer separate regime labels to be compared one by one. They become different ways of drawing the governance boundary between capacity that is locally protected and capacity that is centrally pooled. The model leaves that boundary open. It does not build in a preferred regime; instead, the selected boundary is allowed to vary with demand conditions, coordination burden, and the value of local access under pressure.

### 3.4. The Two-Ward Canonical Case

Although the general formulation allows for n wards, the analytical discussion starts with two. The reason is not that hospital bed systems are this simple. More wards would introduce additional demand correlations, allocation combinations, and boundary cases before the basic governance tension is visible. With two wards, the first trade-off remains visible. The case shows how local access, pooled capacity, and the coordination work needed for reassignment can start to move in different directions, even before the setting becomes more complex.

Pooling across wards needs more than a single unit. In a case with two wards, the basic tension already appears when one ward comes under pressure while another still holds usable capacity. This structure is sufficient to show the pooling opportunity, but also sufficient to raise the governance question: how much capacity should remain locally accessible? The same setting can also represent synchronized demand shocks, protected local reserves, and the coordination burden created when capacity must be reassigned through a central process.

The two-ward case should therefore be read as a device for clarifying the mechanism, not as a claim that hospital bed systems are inherently two-ward. Its role is to keep the trade-off visible before the analysis moves back to richer multi-ward settings. The computational experiments later examine systems with richer ward structures, heterogeneous capacities, and correlated demand patterns to examine whether the same boundary mechanism remains visible beyond the canonical case.

## 4. Model Formulation

### 4.1. Feasible Allocation Under a Governance Boundary

Section 3 introduced α as the governance boundary between protected ward capacity and centrally pooled capacity. We now translate that boundary into the set of effective capacity assignments available after ward-level demand is observed. The formulation is stated for the general *n*-ward system, although the analytical discussion later uses a two-ward canonical case for clarity. This keeps the regime comparison internally consistent and allows the computational experiments to return to larger ward systems.

Operationally, the feasible allocation set describes the assignments available to the bed-management team after demand is observed, given the governance boundary chosen in advance.

Protected local reserves remain immediately available to their own wards, whereas only the pooled portion can be redirected through hospital-wide coordination. In this formulation, X(α) denotes the effective capacity assignments that the chosen governance design permits after demand is observed. It should not be interpreted as physical bed movement. The clinical usability of these assignments varies across scenarios. The frictionless benchmark assumes that the permitted assignments can be fully converted into usable capacity, while the later costly and stress-state settings allow that conversion to be limited by placement judgment, staffing, specialty requirements, and authorization. Let

$$x(D,\alpha )=(x_{1}(D,\alpha ),\ldots ,x_{n}(D,\alpha ))$$

denote the effective capacity assigned across wards in demand state D. Using the reserve and shared-pool definitions in Equation (2), the feasible allocation set is

(3) $$X(\alpha )=\left\{x\in R_{+}^{n}:x_{i}\geq r_{i}(\alpha ),\sum_{i=1}^{n}\;\left[x_{i}-r_{i}(\alpha )\right]\leq S(\alpha )\right\}.$$

The lower-bound constraint $x_{i}\geq r_{i}(\alpha )$ reflects the protected reserve held by ward *i*. This reserve is a local claim on existing capacity, not a requirement that the ward keep beds idle. The shared-pool constraint limits how much capacity can be assigned above the protected reserves. Together, the two constraints translate the governance boundary α into an allocation set.

The endpoints of X(α) recover the two polar regimes. When α=0, all nominal capacity remains locally protected. Since $r_{i}(0)=B_{i}$ and S(0)=0,

(4) $$X(0)=\left\{x\in R_{+}^{n}:x_{i}=B_{i},i=1,\ldots ,n\right\}.$$

This is the allocation set under full decentralization. When α=1, no ward-specific reserve is protected. Since $r_{i}(1)=0$ and S(1)=B,

(5) $$X(1)=\left\{x\in R_{+}^{n}:\sum_{i=1}^{n}\;x_{i}\leq B\right\}.$$

This is the allocation set under full centralization.

For intermediate values 0<α<1, the same feasible-set formulation describes bounded centralization: part of each ward’s nominal capacity is protected, while the rest is pooled. Bounded centralization is therefore not an additional allocation rule, but an interior governance boundary within the same α-framework. Because protected reserves restrict reassignment, full centralization weakly expands the feasible set:

(6) X(α)⊆X(1),∀α∈[0,1].

This inclusion anchors the frictionless pooling benchmark in Section 5. It is a feasibility statement made before coordination friction and tail-risk considerations are introduced. It should therefore be read as the strongest case for full centralization, not as a conclusion that full centralization is always preferred.

### 4.2. Ward-Level Outcomes and Realized Operating Loss

We next translate each feasible capacity assignment into ward-level outcomes. For a realized demand vector D and a feasible allocation x∈X(α), the admitted demand, idle capacity, and unmet demand of ward i are

(7) $$A_{i}(x,D)=min\{D_{i},x_{i}\},{\;\;U}_{i}(x,D)=(x_{i}-D_{i}{)}^{+},{\;\;L}_{i}(x,D)=(D_{i}-x_{i}{)}^{+},$$

where $\left(z{)}^{+}=max\{z,0\right\}$. These definitions decompose the immediate consequences of an allocation into served demand, unused ward-assigned capacity, and demand that cannot be accommodated. Because the feasible set does not require the shared pool to be fully assigned in every demand state, we also record unused shared-pool capacity:

$$P(x,\alpha )=S(\alpha )-\sum_{i=1}^{n}\;[x_{i}-r_{i}(\alpha )].$$

By feasibility, P(x,α)≥0. This term represents pooled capacity that remains operationally unassigned in the realized demand state. It is distinct from ward-level idle capacity $U_{i}(x,D)$, which measures capacity assigned to ward i but not used by demand in that ward. The realized operating loss under allocation x in demand state D is

(8) $$l(x,D;\alpha )=\sum_{i=1}^{n}\;p_{i}L_{i}(x,D)+\sum_{i=1}^{n}\;h_{i}U_{i}(x,D)+\bar{h}P(x,\alpha )+cQ(x,D,\alpha ).$$

where $\bar{h}=n^{-1}\sum_{i=1}^{n}\;h_{i}$. The first term captures shortage, the second term captures ward-level idle capacity, the third term captures pooled capacity that remains available but unassigned, and the fourth term captures coordination burden. We leave Q(x,D,α) general for the moment and define it in Section 4.3.

For a fixed governance boundary α, the second-stage allocation is defined by

(9) $$x(D,\alpha )\in arg\underset{x\in X(\alpha )}{min}\;l(x,D;\alpha ).$$

This formulation makes the response to realized demand explicit. It represents the best feasible response under a given governance boundary, not a literal claim that managers solve a full optimization problem in real time for every demand realization. In the computational experiments, this response is operationalized by the scenario-level allocation procedure described in Algorithm A1, which uses the same feasible-boundary structure and cost components as the analytical model. The algorithm is therefore the numerical implementation of this best-response logic, not a separate modeling assumption. Once x(D,α) is obtained from Equation (9), we write $A_{i}(D,\alpha )$, $U_{i}(D,\alpha )$, $L_{i}(D,\alpha )$, and P(D,α) as shorthand for the corresponding functions evaluated at x(D,α).

### 4.3. Coordination-Friction Specifications

The realized loss in Equation (8) includes $Q\left(x,D,\alpha \right)$, which represents the coordination burden that arises when capacity is used across ward boundaries. In practice, this burden may arise from communication, approval, placement adjustment, specialty compatibility, or delay. We do not model these processes separately. Instead, Q is a reduced-form measure of the coordination intensity implied by the realized allocation.

Using this reduced-form term does not imply that coordination burden arises from a single operational source. In a hospital bed-management setting, the difficulty of using pooled capacity may take several forms. It may increase when the final bed assignment departs substantially from the ward’s usual capacity base. It may also increase when more shared-pool capacity must be activated. In other situations, the main burden may come from the number of wards involved in the reassignment process, or because some wards are clinically and organizationally harder to reassign to or from than others. We therefore use $Q_{1}$ as the main specification and retain $Q_{2}$–$Q_{4}$ as alternative readings of the same coordination problem.

The baseline specification measures coordination friction through realized reallocation intensity, rather than through the formal governance label:

(10) $$Q_{1}(D,\alpha )=\frac{1}{2}\sum_{i=1}^{n}\;\left|x_{i}(D,\alpha )-B_{i}\right|.$$

$Q_{1}$ measures the total deviation of the realized allocation from the nominal capacity vector $B=(B_{1},\ldots ,B_{n})$. The factor 1/2 avoids counting the same reallocation twice. Under full decentralization, $x_{i}(D,0)=B_{i}$ and therefore $Q_{1}(D,0)=0$. Under full centralization, $Q_{1}$ depends on the allocation chosen after demand is observed. FC therefore incurs coordination burden only when the realized allocation involves cross-ward adjustment, not simply because the beds are centrally governed.

Unless stated otherwise, $Q_{1}$ in Equation (10) is used as the baseline measure. This choice charges all regimes for realized departures from nominal ward capacity, not for their formal labels. To check that the analysis is not tied too closely to $Q_{1}$, we also define three additional specifications. Let

$$y_{i}(D,\alpha )=x_{i}(D,\alpha )-r_{i}(\alpha )$$

denote the amount of capacity assigned to ward i from the shared pool. By the definition of X(α), $y_{i}(D,\alpha )\geq 0$ and $\sum_{i}\;y_{i}(D,\alpha )\leq S(\alpha )$.

If coordination burden is closer to the amount of pooled capacity actually used than to the distance from nominal ward capacity, the second specification is:

(11) $$Q_{2}(D,\alpha )=\sum_{i=1}^{n}\;y_{i}(D,\alpha ).$$

A different interpretation is that coordination becomes difficult when more wards have to be brought into the process. Even a moderate amount of shared capacity may require additional negotiation and handoff work when it is spread across several wards. This gives:

(12) $$Q_{3}(D,\alpha )=\sum_{i=1}^{n}\;1\{y_{i}(D,\alpha )>0\}.$$

Finally, reassignment difficulty may vary across wards. For example, intensive care, surgical, infection control, or other highly specialized wards may impose staffing, clinical fit, and authorization requirements that differ from those of a general medical ward. To allow for this heterogeneity, we use:

(13) $$Q_{4}(D,\alpha )=\frac{1}{2}\sum_{i=1}^{n}\;\omega_{i}\left|x_{i}(D,\alpha )-B_{i}\right|.$$

where $\omega_{i}\geq 0$ is a ward-specific reassignment weight. Table 2 summarizes the interpretation of the four coordination-friction specifications.

These specifications should be read as simplified ways of representing coordination burden, not as calibrated estimates of the true cost of hospital bed reassignment. More modestly, they allow us to ask whether the model’s boundary effect depends on one particular definition of coordination friction. In an empirical application, the relevant form of Q would need to be informed by hospital data, such as bed-assignment records, transfer timestamps, approval delays, staffing availability, and specialty compatibility rules.

### 4.4. Tail-Risk Exposure and Governance Performance

Once the allocation x(D,α) has been determined, we evaluate a governance boundary by the operating loss it generates across demand states. For brevity, write Q(D,α) for Q(x(D,α),D,α). We define

(14) $$C_{avg}(\alpha )=E\left[\sum_{i=1}^{n}\;p_{i}L_{i}(D,\alpha )+\sum_{i=1}^{n}\;h_{i}U_{i}(D,\alpha )+\bar{h}P(D,\alpha )+cQ(D,\alpha )\right].$$

This measure aggregates the average operating-loss components under a boundary: unmet demand, unused capacity, unused shared-pool capacity, and coordination effort.

Average loss alone does not distinguish mild shortages from severe system-wide shortages. This distinction matters for bed management because simultaneous shortages across wards place greater pressure on hospital-wide coordination. We therefore define the total system shortage as

(15) $$L_{sys}(D,\alpha )=\sum_{i=1}^{n}\;L_{i}(D,\alpha ).$$

The tail-loss component is defined as

(16) $$C_{tail}(\alpha )=E\left[{\left(L_{sys}(D,\alpha )\right)}^{\gamma }\right],\gamma >1.$$

The exponent γ gives disproportionate weight to larger shortage realizations. We use $C_{tail}(\alpha )$ as a compact proxy for exposure to adverse shortage states, not as a full measure of operational resilience. For the mechanism studied here, this proxy is sufficient because the model asks whether protected local capacity can reduce exposure to severe-shortage states. It does not attempt to measure broader organizational dimensions of resilience, such as recovery speed, staffing flexibility, or emergency expansion capacity.

The resulting governance performance measure is

(17) $$\Pi (\alpha )=-C_{avg}(\alpha )-\eta C_{tail}(\alpha ),\eta \geq 0.$$

Because $C_{avg}$ and $C_{tail}$ are loss terms, a larger value of Π(α) indicates better governance performance. The parameter η controls the weight placed on adverse shortage states. When η=0, the criterion reduces to expected operating loss. When η>0, severe system-wide shortages also enter the evaluation.

### 4.5. The Governance Boundary Problem

The preceding definitions allow us to state the boundary-design problem. The centralization degree α is a planning-level governance choice, not a real-time dispatching variable. Once the boundary is set, demand is realized, and bed capacity is allocated within the feasible set implied by that boundary. The boundary-design problem can be written as

(18) $$\alpha^{*}\in arg\underset{\alpha \in [0,1]}{max}\;\Pi (\alpha ).$$

Any selected optimizer $\alpha^{*}$ identifies where the governance boundary is drawn between locally accessible capacity and hospital-wide pooled capacity. If an optimizer lies at α=0, the preferred boundary corresponds to full decentralization. If an optimizer lies at α=1, the preferred boundary corresponds to full centralization. If an optimizer lies in the interior, $\alpha^{*}\in (0,1)$, the selected design is bounded centralization: part of the ward-level capacity remains locally protected, while the rest is opened to hospital-wide pooling.

This formulation does not impose a preference for bounded centralization. The preferred boundary is determined by the demand process, shortage and idle-capacity weights, coordination friction, and the weight placed on adverse shortage states. The optimum may therefore occur at either endpoint or at an interior value. Equation (18) above defines the boundary problem in mathematical terms. Before turning to the analytical results, it is useful to make its managerial interpretation explicit. Section 4.6 first explains the main parameters in hospital-management terms. Section 4.7 then links the formal components of the model to clinical and operational bed-management processes. These two subsections provide the interpretation needed for the analytical comparison in Section 5.

### 4.6. Practical Interpretation of Key Parameters

The model uses a small set of parameters to represent several managerial features of hospital bed governance. Some of these parameters, such as the centralization degree α, describe how the bed stock is organized before demand is realized. Others, such as c and δ, describe the operating conditions under which pooled capacity is actually used. The parameters η and γ describe how the evaluation weights severe-shortage states relative to average operating loss. Accordingly, these parameters should be read as reduced-form representations of governance design, coordination burden, and tail-risk concern. They are not calibrated estimates for a specific hospital in this study.

Table 3 summarizes the hospital-management interpretation of the main parameters.

Read together, the parameters in Table 3 describe more than the size of a shared pool. The boundary α decides how much capacity is exposed to central coordination, but those beds still have to pass through placement work before they can help a ward in real time. The parameter c attaches weight to that work. The parameters η and γ make severe-shortage states matter in the objective, and δ represents the possibility that pooled capacity is harder to use when several wards are under stress. The model therefore treats pooling as valuable capacity only after it can be identified, coordinated, and turned into a clinically workable placement.

### 4.7. Clinical and Operational Mapping of the Model

The model has deliberately compressed many clinical details into a small number of terms. That compression is useful for analysis, but the terms still need to be read against the bed management work they stand for. Table 4 provides that link. It shows how demand states, pooled capacity, reassignment work, staffing limits, and stress related usability losses can be interpreted in hospital operations. The mapping is not meant to turn the model into a full clinical pathway description. Its purpose is to make clear which parts of bed management are being represented when the formal framework is used to study the boundary between central pooling and local protection.

Table 4 is intended to keep a simple operational distinction visible. A pooled bed does not become usable merely because it appears in the formal pool. Staff must be available, the patient’s service needs must fit the receiving ward, placement approval must be secured, and the transfer still has to be carried out. When any of these steps slows, the bed may remain on the central list while the ward facing shortage receives little immediate relief. In the frictionless benchmark, capacity kept within ward access reduces the shared pool and can therefore look inefficient. Under pressure, the same capacity may work differently. Local routines can make it easier to act quickly, limit placement delay, or prevent admission saturation from worsening. The model does not follow the full clinical pathway behind each placement. The mapping instead clarifies how these operating frictions shape the governance choice between centralized pooling and local protection.

## 5. Analytical Results and Mechanism Development

### 5.1. Frictionless Pooling Benchmark

We first consider an idealized pooling benchmark in which the shared bed stock can be used without operational loss. This benchmark gives full centralization its strongest case: beds are perfectly substitutable across wards, reassignment is immediate after demand is observed, and centralized allocation creates no delay, mismatch, or coordination burden. Because the benchmark is intentionally strong, the standard pooling logic should apply: if all beds can be allocated centrally, the hospital has a larger feasible allocation set than it would have when part of the bed stock remains locally protected.

The benchmark should therefore be read narrowly. It does not assume that all hospital beds are interchangeable in routine clinical practice. Rather, it describes an upper-bound case in which the beds considered for pooling are sufficiently standardized, clinically compatible, and supported by placement rules that allow demand to be absorbed across ward boundaries. Such an approximation may be reasonable for some general medical beds or low-acuity overflow arrangements, but it is far less appropriate for beds tied to specialized equipment, infection-control requirements, dedicated nursing skills, or specialty-specific clinical responsibility, such as ICU, isolation, postoperative specialty, pediatric, maternity, or psychiatric beds. Beginning with this strong case gives full centralization its clearest possible advantage. The later introduction of coordination friction and stress-state pooled-allocation imperfection then examines how the conclusion changes once the conversion from formal pooled capacity to usable ward-level capacity becomes less immediate.

Formally, consider a frictionless pooling environment. The central planner observes the realized demand vector D before making the second-stage allocation decision. Beds are fully substitutable across wards, and assigning capacity away from its nominal ward does not create clinical mismatch or service-quality loss. Reassignment is immediate and costless, so coordination friction is absent, c=0. The realized loss therefore contains only shortage and idle-bed costs:

(19) $$l_{0}(x,D)=\sum_{i=1}^{n}\;p_{i}L_{i}(x,D)+\sum_{i=1}^{n}\;h_{i}U_{i}(x,D),$$

where $L_{i}(x,D)$ and $U_{i}(x,D)$ denote, respectively, unmet demand and idle capacity for ward *i* under allocation x. For a given centralization degree α, define the realized benchmark loss as

$$l_{0}^{\alpha }(D)=\underset{x\in X(\alpha )}{min}\;l_{0}(x,D).$$

Let $\Pi_{0}(\alpha )=-E[l_{0}^{\alpha }(D)]$ denote the corresponding benchmark performance under centralization degree α. The benchmark suppresses coordination and tail-risk terms to isolate the classical pooling effect. Let $X^{FC}$ denote the feasible allocation set under full centralization, and let X(α) denote the feasible allocation set under centralization degree α∈[0,1].

**Theorem** **1.** *Frictionless FC dominance.*

Under the frictionless pooling benchmark,

(20) $$\Pi_{0}(1)\geq \Pi_{0}(\alpha ),\forall \alpha \in [0,1].$$

That is, full centralization weakly dominates any bounded-centralization regime under the benchmark loss $l_{0}$.

The proof follows from feasible-set inclusion. Under the formulation in Section 4, bounded centralization restricts how the fixed bed stock can be assigned; it does not create allocations that FC could not implement. For any α∈[0,1], every allocation feasible under X(α) is also feasible under full centralization by (6). Hence, for every realized demand vector D,

(21) $$\underset{x\in X^{FC}}{min}\;l_{0}(x,D)\leq \underset{x\in X(\alpha )}{min}\;l_{0}(x,D).$$

Taking expectations over D and using $\Pi_{0}(\alpha )=-E[l_{0}^{\alpha }(D)]$, gives Equation (20).

**Figure 2:**
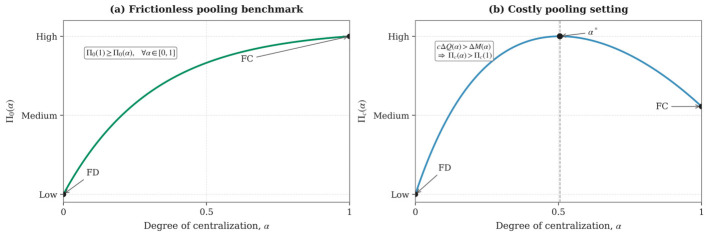
Performance over the governance boundary under frictionless and costly pooling. Panel (**a**) shows the frictionless reference case. When pooled beds can be reassigned without delay, mismatch, or coordination burden, the performance curve reaches its highest value at full centralization. Panel (**b**) incorporates the cost of using the shared pool. The asterisk denotes the performance-maximizing governance boundary $\alpha^{*}$ In this case, the curve may bend back before α reaches 1, because a larger formal pool can also create more work in turning pooled capacity into usable ward-level capacity. The curves are normalized to highlight this change in mechanism, rather than to report calibrated performance levels for a particular hospital.

Theorem 1 provides the reference point for the rest of the analysis. When pooling is costless, timely, and operationally neutral, local protection does not improve on full centralization in the benchmark shortage–idle loss. In hospital-management terms, this is the case in which pooled beds can be converted into usable ward-level capacity without delay, mismatch, or coordination burden. Figure 2a illustrates this benchmark mechanism, under frictionless pooling, benchmark performance reaches its highest value at α=1. The next question is how this conclusion changes when the use of pooled capacity carries operational burden.

### 5.2. Costly Pooling and the Erosion of FC Dominance

Theorem 1 rests on a demanding premise, the flexibility created by full centralization can be used at no cost. In hospital bed governance, central reassignment may involve communication, capacity search, approval, patient-placement decisions, and coordination across ward boundaries. The issue is not only whether capacity is formally pooled, but also whether pooled capacity can be converted into usable ward-level capacity without additional burden. Once these activities carry operational burden, feasible-set inclusion alone no longer determines the comparison. Let

$$M(D,\alpha )=\sum_{i=1}^{n}\;p_{i}L_{i}(D,\alpha )+\sum_{i=1}^{n}\;h_{i}U_{i}(D,\alpha )$$

denote the shortage–idle component of realized loss before coordination burden is added. With positive coordination friction,

(22) $$l_{c}(D,\alpha )=M(D,\alpha )+cQ(D,\alpha ),c>0,$$

where Q(D,α) captures realized coordination burden. The corresponding performance measure is

(23) $$\Pi_{c}(\alpha )=-E\left[M(D,\alpha )+cQ(D,\alpha )\right].$$

The key modeling point is that Q(D,α) is not a fixed surcharge imposed on FC. It is a reduced-form measure of realized coordination intensity. Under the baseline specification $Q_{1}$ in Equation (10), FC incurs coordination burden only when the implemented allocation involves cross-ward adjustment. BC is treated under the same rule: it is not rewarded for being an interior design and improves on FC only in cases where the reduction in realized coordination effort is large enough to outweigh the additional shortage–idle loss.

**Proposition** **1.** *Costly pooling can erode FC dominance.*

For a given α∈(0,1), define

(24) $$\Delta M\left(\alpha \right)=E\left[M\left(D,\alpha \right)-M\left(D,1\right)\right],\;\Delta Q(\alpha )=E[Q(D,1)-Q(D,\alpha )].$$

The first term $\Delta M\left(\alpha \right)$ records how much additional shortage–idle loss the boundary α carries relative to FC. The second, ΔQ(α), records the expected reduction in coordination burden relative to FC. If that reduction is positive and large enough to cover the additional shortage–idle loss,

(25) $$\Delta Q\left(\alpha \right)>0,\;\;c\Delta Q(\alpha )>\Delta M(\alpha ),$$

then

(26) $$\Pi_{c}(\alpha )>\Pi_{c}(1).$$

In that case, the feasible-set advantage of FC no longer settles the comparison once realized coordination burden is included.

The proof follows directly from the definition of $\Pi_{c}$. Subtracting $\Pi_{c}(1)$ from $\Pi_{c}(\alpha )$ gives

(27) $$\Pi_{c}(\alpha )-\Pi_{c}(1)=-\Delta M(\alpha )+c\Delta Q(\alpha ).$$

Hence, if cΔQ(α)>ΔM(α), then $\Pi_{c}(\alpha )-\Pi_{c}(1)>0$, and the result follows.

This result is deliberately limited. It does not rank BC above FC in general. It shows only that a bounded regime can outperform FC when the coordination effort saved from using less pooled reassignment is larger than the shortage–idle loss created by restricting the feasible allocation set. For hospital managers, the point is not that a smaller pool is better, but is that pooled capacity may have to be coordinated before it becomes usable, and that this cost can affect where the governance boundary is drawn. Figure 2b illustrates this departure from the frictionless benchmark: with positive coordination friction, the performance curve need not peak at full centralization.

### 5.3. Local Protection as Resilience Capacity

Coordination friction explains why the use of the shared pool may be costly. However, cost is not the only reason why the degree of pooling matters. Full pooling also reduces immediate ward-level access. These are separate channels: one concerns the burden of using pooled capacity; the other concerns the value of retaining capacity that can be reached locally under pressure. Under bounded centralization, ward *i* retains $r_{i}(\alpha )$ as protected local capacity. This should not be read as a preference for keeping capacity idle, but as a local claim on capacity that can be used before the ward depends on central reassignment.

Under the frictionless benchmark in Theorem 1, this channel is intentionally absent. If reassignment is immediate, reliable, and costless, FC can replicate any bounded arrangement. There is then no separate advantage to keeping capacity locally protected. Local protection begins to matter only when access to pooled capacity is imperfect, time-sensitive, or costly under adverse states.

**Figure 3:**
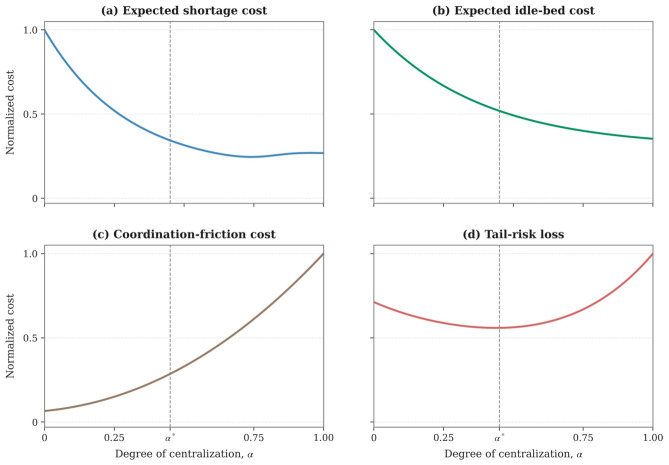
Cost-component decomposition over the centralization boundary. The figure decomposes the normalized performance curve into expected shortage cost, expected idle-bed cost, coordination-friction cost, and tail-risk loss. The vertical dashed line marks the maximizing interior boundary $\alpha^{*}$. The decomposition is included because an interior boundary is not produced by a single cost component. At lower values of α, expanding the shared pool can reduce mismatch between wards. At higher values, the marginal pooling gain may become smaller, while the system relies more heavily on coordinated reassignment and retains less immediately accessible local capacity. Components are normalized to show their relative movement, rather than calibrated hospital-level magnitudes.

This mechanism becomes most visible when ward demands move together. Pooling is most valuable when high demand in one ward can be offset by slack in another. As ward-level demand shocks become more synchronized, this offsetting logic weakens. The shared pool may face simultaneous claims, while protected local capacity can provide immediate ward-level absorption. Using Equations (15) and (16), this channel enters the model through total system shortage $L_{\mathrm{sys}}(D,\alpha )$ and the tail-risk component $C_{\mathrm{tail}}(\alpha )$, where γ>1 places disproportionate weight on severe-shortage realizations. This adverse-state channel is reflected in the tail-risk component shown later in Figure 3d.

**Mechanism** **Proposition** **2.** *Local protection and tail exposure.*

Suppose ward demands are positively correlated, shortage losses are convex, and centralized reassignment is costly or delayed in adverse demand states. Protected local capacity may reduce exposure to severe simultaneous shortage states. In such parameter regions, bounded centralization can provide resilience value that is not captured by average shortage–idle loss alone.

The mechanism is conditional. Local protection helps only when the value of immediate ward-level absorption outweighs the loss of some pooling flexibility. If reassignment is frictionless or demand shocks are weakly synchronized, the pooling benchmark in Theorem 1 remains the natural reference point.

Proposition 1 and Mechanism Proposition 2 identify two distinct departures from the frictionless benchmark. The first concerns the cost of using pooled capacity, the second concerns the loss of protected local access in adverse states. Together, they imply that the effect of α on overall performance may be non-monotone.

### 5.4. Interior Governance Boundary

We now return to the full performance measure in Equation (17). Increasing α expands the shared pool, but it also changes coordination burden and the amount of protected local capacity. Accordingly, the effect of centralization depends on the balance among shortage–idle loss, coordination friction, and tail-risk exposure.

This balance can vary across the centralization range. Near α=0, increasing α enlarges the shared pool and may sharply reduce cross-ward mismatch. Near α=1, additional pooling may bring smaller marginal gains while further reducing protected local reserves and increasing reliance on central reassignment. Together, these forces can make the performance curve non-monotone.

**Proposition** **3.** *Interior governance boundary.*

When coordination friction is positive and protected local capacity has adverse-state value, Π(α) need not be monotone on [0,1]. There may exist parameter regions in which

(28) $$\alpha^{*}\in (0,1).$$

A sufficient condition for such an interior boundary is that there exists $\bar{\alpha }\in (0,1)$ such that

(29) $$\Pi \left(\bar{\alpha }\right)>\Pi \left(0\right),\;\;\Pi (\bar{\alpha })>\Pi (1),$$

Then the preferred governance boundary cannot be represented by either full decentralization or full centralization alone; it corresponds to a bounded design that combines hospital-wide pooling with protected local access.

This result is conditional and does not require Π(α) to be globally concave or the interior maximizer to be unique. It states only that, under some operating environments, selective pooling combined with protected local capacity can outperform both extremes. Figure 3 decomposes this pattern into shortage, idle-bed, coordination-friction, and tail-risk components.

### 5.5. Computational Implications

The analytical results above do not provide a universal ranking of FD, BC, and FC. Instead, they specify the conditions under which the frictionless dominance of FC may be weakened: realized coordination burden, adverse-state exposure, and the loss of immediate local access. These mechanisms define the role of the computational experiments.

Accordingly, the computational experiments in Section 6 are used as mechanism checks rather than calibrated hospital-level predictions. They examine whether the interior-boundary pattern remains visible as the operating environment changes.

The experiments serve three roles. First, they verify the frictionless benchmark by checking whether FC remains performance-consistent when pooled allocation is fully effective and costless. Second, they examine whether coordination friction alone can produce a substantive improvement over FC. Third, they test whether an interior boundary becomes more meaningful when pooled allocation becomes less reliable in high-pressure states.

Finally, an interior numerical maximizer should not automatically be interpreted as evidence against FC. Section 6 therefore distinguishes between a raw interior maximizer and a substantively meaningful improvement over FC. This distinction is important for reading the computational results because an inward shift in the boundary is not necessarily managerially meaningful. Its relevance depends on whether the performance gain over FC is large enough to be substantive.

## 6. Computational Experiments

### 6.1. Experimental Design and Parameterization

The computational experiments are used to check the proposed mechanisms under controlled assumptions, not to forecast bed-management outcomes for a specific hospital. They compare how the governance boundary α behaves as the ideal pooling benchmark is gradually relaxed, moving from frictionless pooling to costly pooling and then to stress states in which pooled reassignment becomes less reliable. This structure makes the scenarios transparent and keeps the interpretation limited. The simulations illustrate when an interior boundary can emerge; they do not identify the optimal α for any particular hospital.

We begin with the two-ward canonical setting introduced in Section 3. A one-ward system cannot generate a cross-ward pooling problem, while a larger multi-ward system would introduce heterogeneity before the baseline mechanism is visible. For this baseline, each ward is assigned the same nominal capacity, $B_{i}=50$, so that $B=\left(50,50\right)$. The scale is not meant to reproduce the bed stock of a particular hospital department. It gives a transparent setting in which changes in the governance boundary can be examined before capacity heterogeneity is introduced.

Demand pressure is expressed as $\kappa_{i}=\mu_{i}/B_{i}$. In the baseline setting, $\kappa =\left(0.96,0.92\right)$, giving $\mu =\left(48,46\right)$. The baseline demand levels are chosen so that both wards operate close to nominal capacity, but not under chronic overload. This leaves room for governance design to show an effect. If demand were much lower, neither pooling nor local protection would have much operational consequence in the experiment. With demand well above capacity, every governance boundary would perform poorly. The chosen baseline therefore leaves room for pooling to reduce mismatch while still allowing coordination burden and local protection to matter. Demand variability is set at cv=0.17, and the baseline correlation is ρ=0.4. This gives a moderate level of uncertainty and demand synchronization: ward pressures are related enough for pooled capacity to be contested in some states, but not so strongly synchronized that the pooling benchmark is undermined by construction. Demand scenarios are then generated from a nonnegative transformation of a multivariate normal vector:

(30) $$\tilde{D}∼N(\mu ,\Sigma ),D_{i}=max\{0,{\tilde{D}}_{i}\},$$

Thus, μ should be interpreted as the pre-truncation demand level used in scenario generation rather than the exact mean of the transformed nonnegative demand distribution. The cost parameters are chosen to keep the comparison interpretable in clinical-operational terms rather than as monetary estimates. Shortage costs are $p=\left(10,12\right)$, while idle-capacity costs are $h_{i}=1$. This assigns a substantially higher penalty to unmet bed demand than to unused assigned capacity, consistent with the governance focus of the paper: the main concern is not merely whether a bed is unused, but whether admission pressure cannot be absorbed in time. The small difference between the two ward-level shortage penalties introduces limited heterogeneity, without making the experiment depend on a dominant ward.

The baseline coordination-friction weight is c=2.0. This value is used as a moderate reference level, not as a calibrated estimate of transfer delay or administrative cost. Its role is to make the coordination burden visible while allowing the robustness checks to show how the classification changes when this burden is weaker or stronger. The same logic applies to the tail-risk terms. We set η=0.05 and γ=2 so that severe-shortage states are represented in the performance measure, but do not overwhelm the average shortage, idle-capacity, and coordination components. The main simulations use N=12,000 demand scenarios. This sample size is large enough to stabilize the reported baseline comparisons, and the larger-sample checks reported in Table 5 are used to verify that the qualitative classifications are not driven by a particular simulation draw.

Stress-state pooled-allocation imperfection is introduced after the baseline environment has been specified. We define stress states as the upper 15% of total demand realizations, because the concern is not ordinary variation around the mean but the upper-tail situations in which several wards may be trying to draw on the shared pool at the same time. In those states, a centrally pooled bed may still be visible in the system, but the work of converting it into usable ward-level capacity can become more difficult. We therefore set

(31) $$\lambda_{s}=max\left\{\lambda_{min},1-\alpha \delta (1+\rho )1_{s\in S^{stress}}\right\}.$$

Here, $\lambda_{min}=0.94$ sets a lower bound on the usable share of pooled capacity, and the baseline stress-imperfection case uses δ=0.02. In these experiments, the scenario-level allocation set is obtained from $X\left(\alpha \right)$ by replacing the nominal shared-pool capacity $S\left(\alpha \right)$ with the usable shared-pool capacity $\lambda_{s}S\left(\alpha \right)$. The adjustment therefore changes the operational usability of pooled capacity in stress states, not the physical bed stock. When δ=0, pooled allocation remains fully effective. When δ>0, pooled capacity becomes less readily converted into usable ward-level capacity under synchronized high-pressure states, for example because approval, placement judgment, patient-ward fit, and competing ward claims become more difficult to resolve in time. The baseline value is deliberately mild, so that the experiment tests whether an interior boundary can appear without assuming a large failure of pooling.

For each α, simulated governance performance is computed as

(32) $$\hat{\Pi }(\alpha )=-\frac{1}{N}\sum_{s=1}^{N}\;\left[\sum_{i}\;p_{i}L_{is}(\alpha )+\sum_{i}\;h_{i}U_{is}(\alpha )+\bar{h}P_{s}(\alpha )+cQ_{s}(\alpha )+\eta (L_{s}^{sys}(\alpha ){)}^{\gamma }\right].$$

We evaluate performance over the grid

(33) A={0,0.02,…,1}.

To distinguish a raw interior maximizer from a substantively meaningful improvement over FC, we define

(34) $$\Delta_{BC-FC}=\underset{\alpha \in (0,1)}{max}\;\hat{\Pi }(\alpha )-\hat{\Pi }(1).$$

An interior boundary is classified as substantive BC only if

(35) $$\Delta_{BC-FC}>\epsilon_{\Delta }.$$

The baseline analyses use $\epsilon_{\Delta }=0.5$, and alternative thresholds are examined in the robustness checks. If the raw maximizer is interior but $\Delta_{BC-FC}<\epsilon_{\Delta }$, the case is reported as FC-comparable. The threshold is used only for substantive classification. It does not change the simulated performance values or the raw maximizing α. This distinction matters because a numerical maximum can occur at an interior value of α even when the performance difference from FC is practically negligible. The classification rule prevents such cases from being interpreted as substantive evidence against full centralization.

### 6.2. Benchmark Experiments

We compare three benchmark cases that progressively relax the ideal conditions underpinning full centralization. The first is frictionless pooling, in which reassignment is fully effective and carries no coordination cost. The second adds coordination cost while keeping pooled allocation fully effective: central reassignment may be costly, but pooled capacity remains fully usable once assigned. The third adds mild stress-state pooled-allocation imperfection to costly pooling. The sequence is intended to separate the classical pooling effect from the additional question of whether pooled capacity remains usable when reassignment becomes costly or pressure-sensitive.

**Figure 4:**
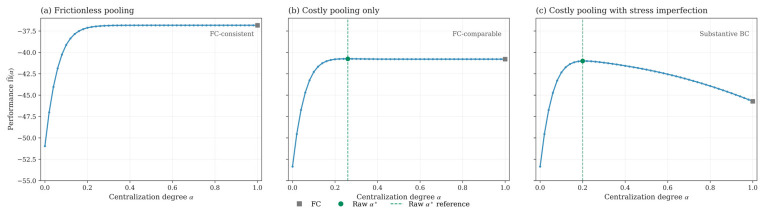
Benchmark performance under progressively relaxed pooling assumptions. Panel (**a**) starts from the frictionless benchmark. Panel (**b**) keeps pooled allocation fully effective but adds coordination friction. Panel (**c**) further allows for a mild loss in pooled-allocation effectiveness during high-pressure states. The panels should therefore be read as a sequence, not as three unrelated examples. The comparison separates a numerical movement of the raw maximizer from a substantively meaningful improvement over full centralization, which is reported using $\Delta_{BC-FC}$ and $\epsilon_{\Delta }=0.5$.

In the third benchmark, a pooled bed can remain visible in the central system but not be usable by another ward in time. As noted earlier, before a transfer can occur, placement staff may still need authorization and a sequence of follow up arrangements. Under system pressure in particular, these processes may slow down or fail to be completed. This gives Equation (31) its operational interpretation. The experiment still keeps the physical pool in place. Pooled beds do not disappear, and they are not treated as wholly unusable. The adjustment is intended to stay mild. It represents delayed conversion rather than the loss of pooled beds themselves. Only the share of nominal pooled capacity that can become effective ward-level capacity under stress is reduced. Figure 4 reports the resulting performance curves.

This sequence leads to three different interpretations. In the frictionless case, FC is performance-consistent: the best interior boundary ties FC but does not improve on it. Adding coordination friction changes the location of the raw maximizer, but not the substantive conclusion. The best raw value is now interior, yet its gain over FC remains below the reporting threshold, so the case is classified as FC-comparable. The interpretation changes only in the third benchmark, where pooled capacity is both costly to use and mildly less reliable in stress states. In that case, the best interior boundary produces a gain above the substantive threshold and is classified as BC. This result should therefore be read as conditional. The experiment does not rank BC above FC in general; it shows that the governance boundary can become meaningful when the formal size of the pool and the clinical-time usability of that pool begin to diverge.

### 6.3. Mechanism Decomposition

**Figure 5:**
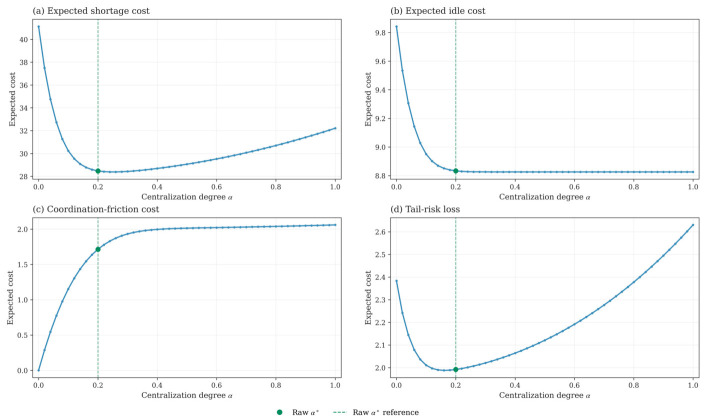
Cost-component decomposition in the baseline stress-imperfection case. The figure separates total simulated loss into expected shortage cost, expected idle-capacity cost, coordination-friction cost, and tail-risk loss. The vertical dashed line marks the raw interior maximizer, $\alpha^{*}=0.20$. This decomposition explains why the interior result in Figure 4c arises. Pooling is beneficial over the first part of the range because it reduces mismatch between wards. The advantage weakens when α becomes high, as the system depends more heavily on centrally reassigned capacity that is costly to coordinate and less reliable under stress. The component values are used to interpret the mechanism, not to provide calibrated cost estimates for a particular hospital.

The benchmark results show when an interior boundary becomes substantive, but the total performance curve does not identify which objective component drives the shift. We therefore decompose the baseline stress-imperfection case into its main cost components. The purpose is not to introduce another performance criterion, but to show how pooling gains, coordination burden, and adverse-state exposure jointly shape the governance boundary. For each value of α, the simulated loss corresponding to (32) can be written as

$$-\hat{\Pi }(\alpha )={\hat{C}}^{short}(\alpha )+{\hat{C}}^{idle}(\alpha )+{\hat{C}}^{Q}(\alpha )+{\hat{C}}^{tail}(\alpha ),$$

where ${\hat{C}}^{short}(\alpha )$ is the expected shortage cost, ${\hat{C}}^{idle}(\alpha )$ is the expected idle-capacity cost, ${\hat{C}}^{Q}(\alpha )$ is the coordination-friction cost, and ${\hat{C}}^{tail}(\alpha )$ is the tail-risk loss. The idle-capacity component includes both unused ward-assigned capacity and unused shared-pool capacity, consistent with the operating-loss definition in Section 4.2. Figure 5 reports these four components for the baseline stress-imperfection case.

The component curves show the same pattern. At low-to-moderate α, pooling reduces shortage and idle capacity, and the interior result should therefore not be interpreted as evidence that pooling is ineffective. The pooling benefit is visible in the first part of the range. As α increases, coordination-friction cost rises, and the system becomes more exposed to reassignment loss in high-demand states. The interior optimum therefore reflects a balance: moderate pooling reduces mismatch, while excessive reliance on the shared pool raises coordination burden and tail-risk exposure.

### 6.4. Boundary Analysis over Coordination Friction and Pooled-Allocation Imperfection

The benchmark value δ=0.02 represents only one way to operationalize the usability adjustment in Equation (31). To check whether the benchmark classification depends on this specific value, we vary both the coordination-friction weight c and the stress-state pooled-allocation imperfection intensity δ. For each pair $\left(c,\delta \right)$, we evaluate $\hat{\Pi }\left(\alpha \right)$ over the grid in Equation (33), compute $\Delta_{BC-FC}$ using Equation (34), and apply the classification rule in Equation (35).

Because the transition near δ=0 is substantively important, we use a refined low-δ grid, δ∈{0,0.0025,0.005,0.0075,0.01,0.015,0.02}, together with c∈{0,1,…,10}. This refinement allows us to distinguish the fully effective pooling case from mild departures from fully usable pooled capacity under stress.

**Figure 6:**
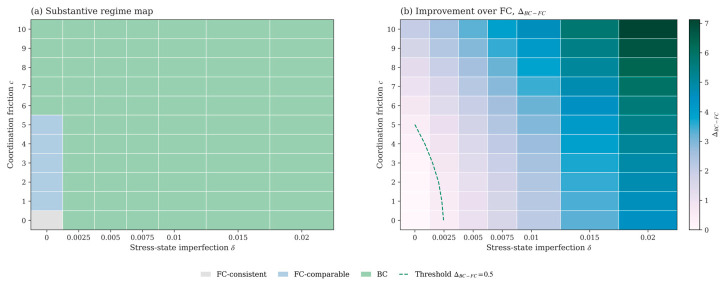
Boundary analysis across coordination friction and stress-state pooled-allocation. Panel (**a**) reports the substantive regime classification over *c* and δ, using the threshold $\epsilon_{\Delta }=0.5$. Panel (**b**) reports $\Delta_{BC-FC}$, with the dashed contour marking $\Delta_{BC-FC}=0.5$. The figure shows how the benchmark classification changes across operating conditions, not to identify calibrated cutoff values for a particular hospital.

Figure 6 shows that the BC classification is not produced simply by choosing an interior governance boundary or by adding coordination friction. When pooled allocation remains fully effective, the cases remain largely FC-comparable, which is consistent with the costly-pooling-only benchmark. The BC region emerges only after the usability loss in stress states is introduced, and only for part of the c–δ space. This distinction matters for interpreting Equation (31). The formula is not being used to make any positive δ automatically favor BC; instead, it identifies the operating region in which the pooling benefit is offset by the combined effect of coordination burden and stress-state conversion loss. The result remains conditional on the simulated high-pressure environment, the severe-shortage penalty, and the substantive-classification threshold.

This pattern raises a more specific robustness question. The result would be less persuasive if it depended mainly on the reporting threshold, a single simulation draw, or a particular measure of coordination burden. We therefore check these issues next before returning to the broader question of whether the same mechanism remains visible in larger ward systems.

### 6.5. Sensitivity and Robustness Checks

The preceding results depend on a classification rule, simulated demand scenarios, a baseline measure of coordination burden. We therefore treat the robustness checks as a way to address a practical question: whether the main interpretation would materially change if the reporting threshold were tightened or relaxed, if the Monte Carlo sample were enlarged, or if coordination friction were measured from a different operational angle. Table 5 reports the threshold and simulation-size checks.

The classifications remain stable under both checks. Varying εΔ changes only the reporting threshold for what counts as a substantive improvement; it does not alter the simulated performance curves or the raw maximizing boundary. Enlarging the Monte Carlo sample leads to the same qualitative reading. The frictionless benchmark remains FC-consistent, the costly-pooling-only case remains FC-comparable, and the mild stress-imperfection case remains stable under both checks.

We also consider whether the interior pattern depends on the baseline definition of coordination friction. The main experiments use $Q_{1}$, which measures realized deviation from nominal ward capacity. This is a natural baseline because it links coordination burden to actual reassignment rather than to the formal name of the governance regime. Coordination work, however, can also be measured from other operational perspectives. Appendix Table A2 repeats the baseline stress-imperfection experiment using the normalized alternatives $Q_{2}$, $Q_{3}$, and $Q_{4}$, which respectively emphasize shared-pool use, the number of wards involved, and ward-specific reassignment difficulty. The raw best boundary remains interior and the substantive classification remains BC under these alternatives. This does not prove robustness to every possible coordination-cost formulation, but it reduces the concern that the finding is an artifact of measuring coordination burden only through $Q_{1}$. The next subsection examines whether the same mechanism remains visible beyond the two-ward setting.

### 6.6. Multi-Ward Extension

The previous experiments use a two-ward system to keep the mechanism transparent. We now examine whether the classification pattern persists in larger systems. The extension considers n = 5 and n = 10 wards with moderate heterogeneity in ward capacity, demand pressure, demand variability, and shortage penalties, while keeping the same allocation logic and α-grid. The extension is used as a mechanism check, not as a calibrated comparison of hospitals with different ward-system sizes.

For each system size, we compare two pooling conditions. The first assumes fully effective pooled allocation. The second adds the same mild stress-state pooled-allocation imperfection used in the benchmark experiments. This comparison separates the effect of reduced pooled-reassignment reliability under pressure. Table 6 reports the results.

Table 6 shows that the multi-ward extension does not automatically favor BC. When pooled allocation is fully effective, the raw maximizer can still lie in the interior, but the gain over FC is negligible. For n=2, 5 and 10, all no-imperfection cases are classified as FC-comparable. This is useful evidence for interpreting the model, adding more wards does not, by itself, make bounded centralization substantively better than FC. When the shared pool works as intended, FC remains a strong benchmark.

**Figure A1:**
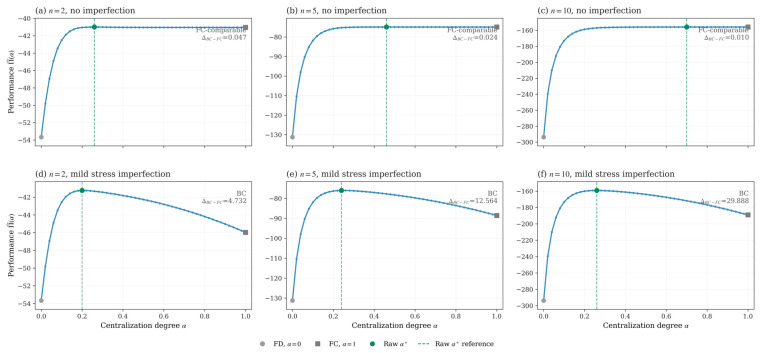
Multi-ward performance curves under fully effective and stress-imperfect pooling. The figure reports $\hat{\Pi }(\alpha )$ over the full centralization grid for multi-ward systems with n=2,5,10. Panels (**a**–**c**) assume fully effective pooled allocation, while panels (**d**–**f**) introduce mild stress-state pooled-allocation imperfection. The green marker and dashed vertical line indicate the raw maximizing boundary $\alpha^{*}$, and the gray square marks full centralization (α=1). In the fully effective pooling cases, the raw optimizer may lie in the interior, but the improvement over FC remains small and is classified as FC-comparable. Under mild stress imperfection, the interior boundary becomes substantively attractive across all three system sizes. Larger $\Delta_{BC-FC}$ values in larger systems partly reflect aggregate system-level cost accounting and should not be interpreted as calibrated hospital-level policy gains.

The pattern changes once mild stress-state imperfection is introduced. In all three systems, the best-performing boundary is interior, and the improvement over FC exceeds the substantive threshold. The exact value of $\alpha^{*}$ varies with system size, as expected, but the classification does not, these stress-imperfection cases are classified as BC. Appendix Figure A1 reports the corresponding full α-grid curves. The visual pattern is consistent with Table 6. Under fully effective pooling, the curves rise quickly and then flatten near FC. Under stress-state imperfection, the curves peak in the interior and then decline as the system approaches full centralization.

These multi-ward results should be read as mechanism checks rather than calibrated policy comparisons across hospital sizes. The larger $\Delta_{BC-FC}$ values for n=5 and n=10 partly reflect aggregate system-level cost accounting. The important point is therefore not the numerical value of $\alpha^{*}$ or $\Delta_{BC-FC}$ in any one system. Rather, it is that the boundary mechanism is not confined to the two-ward case. With fully effective pooling, FC remains performance-comparable, whereas stress-state reassignment loss restores the value of retaining some local protection. This supports the broader view of bounded centralization as a conditional governance design rather than a general substitute for full centralization.

The preceding analyses examine the governance mechanism from several complementary angles, including benchmark scenarios, cost decomposition, boundary analysis, robustness checks, and multi-ward extensions. Table 7 synthesizes these results to show how the qualitative interpretation changes across computational scenarios and how it is shaped by the main robustness checks.

### 6.7. Summary of Computational Findings

The computational results should be read as a sequence of mechanism checks rather than a simple ranking of the three governance regimes. The frictionless benchmark preserves the usual pooling argument: when pooled capacity can be reassigned without loss, FC remains the natural reference point. When coordination friction is added, the performance curve changes and the raw maximizer can move away from α=1. That numerical shift should not be read, on its own, as evidence for a substantively meaningful BC regime. In the costly-pooling-only case, the gain relative to FC remains too small to carry much managerial weight.

When pooled capacity becomes less reliable in stress states, the interior solution should not be judged only by where the maximizer occurs. The cost decomposition gives a clearer reading of what is happening. At low and moderate values of α, pooling still helps: it shifts capacity toward wards with unmet demand and reduces cross-ward mismatch. The trade-off changes as α increases. More of the system’s performance then depends on beds that must pass through central coordination before they can be used, and these beds are exposed to the stress-state usability loss. The interior BC pattern reflects this tension. It emerges when c and δ are high enough to make heavy reliance on the shared pool costly, while pooling still retains enough matching value to be useful.

The robustness checks give little reason to revise this reading. The BC classification in the stress imperfection case is not created by a loose substantive improvement threshold, a small Monte Carlo sample, or the particular baseline choice of $Q_{1}$. Tightening or relaxing the reporting threshold leaves the qualitative classification unchanged, and the larger simulation sample gives the same pattern. Replacing $Q_{1}$ with alternative normalized measures of coordination friction also preserves the interior result. The multi-ward extension points in the same direction. A larger ward system does not make BC substantively better than FC when pooled allocation is still fully effective. The interior pattern reappears only after stress-state reassignment loss is introduced, which keeps the mechanism tied to pooled capacity that becomes harder to use under pressure.

The results therefore call for a conditional reading. They do not show that BC generally dominates FC. They point instead to a more limited operating logic. Bounded centralization becomes substantively attractive when pooling still helps reduce mismatch, while the conversion of pooled capacity into usable capacity becomes costly or less reliable under pressure. For managers, the issue is therefore not simply the size of the shared pool. It is also whether that pooled capacity can be made usable in the demand states where it is most needed.

## 7. Managerial and Policy Implications

### 7.1. Pooling as Usable Capacity, Not Only Formal Capacity

Ward demand seldom rises in the same pattern across the hospital. One unit may have an unused bed while another is trying to keep admissions moving. That unevenness is the basic reason pooling remains important in bed reform. If patients can be placed safely across wards, decisions are made without much delay, and coordination adds little work, a hospital-wide pool gives bed teams the widest room to respond. In that setting, full centralization remains the benchmark.

The difficulty starts once a bed has been counted as part of the pool. It still has to be found, cleared for use, checked against the patient’s needs and the receiving ward’s capacity, and turned into a placement before the demand situation changes. On a quiet day, this work may barely be noticed. When several wards need help at once, it can become the step that slows the whole response. The bed board may still show a large shared pool, but managers may have less usable flexibility than the formal number implies. The practical test is whether shared capacity can be converted into ward-level placements quickly enough when pressure is concentrated across wards.

### 7.2. Coordination Burden and Local Protection as Governance Variables

After demand is observed, Q reflects the work needed to turn an allocation into an actual placement. Full centralization may add little burden when patients can still be placed close to each ward’s usual capacity. A bounded design is not automatically lighter. If several wards become short at the same time, the hospital may have to use pooled beds repeatedly, with each use bringing approval, movement, and placement work. The relevant issue is the demand state that appears, rather than the governance label selected in advance.

The protected reserve $r_{i}(\alpha )$ in Equation (2) should be read in the same operational way, but from the access side. It does not create new beds, and it should not be interpreted as capacity deliberately left empty. It is a priority claim on beds already inside the hospital. Its value lies in reachability. A ward can draw on this capacity through its own admission routines, staffing arrangements, and clinical judgment before waiting for central reassignment. When shared placements are quick and reliable, this advantage may be limited. It matters more when pressure rises across several wards at once, or when central staff cannot identify, authorize, and complete transfers quickly enough.

For bounded centralization to be workable, hospitals need explicit rules around these claims. The shared pool remains available, but some usable capacity is not placed entirely behind the full reassignment process. It stays close enough for wards to use directly as pressure rises. In practice, hospitals would need to define when local claims are protected, when they give way to the shared pool, and who has authority to override them during hospital-wide pressure.

### 7.3. Designing a Data-Informed Boundary for Bed Reform

Hospitals do not have to treat centralization as the inevitable endpoint of bed reform. Before moving more capacity into a shared pool, they can ask what the change is likely to do under their own conditions. Occupancy histories, bed-assignment records, stress scenarios, and simulation runs can all help trace how performance changes as the access boundary moves.

The α framework is useful because it puts full decentralization, bounded centralization, and full centralization on the same scale. They are not three unrelated policy labels, but different positions along a common boundary between ward access and central coordination. This framing shifts the reform question from which regime sounds preferable to where access should actually sit.

The evidence will not always point cleanly to one answer. Full centralization may remain the best design in some hospitals. In other cases, several designs close to α=1 may perform so similarly that the operational difference is small, even if one value ranks first in a simulation. An interior value also needs to be interpreted rather than copied into policy. Its apparent advantage may come from fewer unmet bed requests, less unused capacity, lower coordination work, or reduced exposure to severe-shortage states. Those sources of gain have different managerial meanings.

The useful α is therefore likely to vary across hospitals. Service mix, staffing cover, transfer routines, and placement authority all affect how much shared capacity can be used in practice. Managers need to know which beds a central team can identify and release quickly, which services require stronger local access because reassignment is difficult, and when central authority should expand because protected local access is no longer the safest use of capacity. A defensible boundary should fit observed demand, staffing conditions, and coordination capacity. It should also be revisited as those conditions change.

### 7.4. Limited Centralization in Hospital Practice

In many hospitals, centralization begins with shared visibility rather than a full transfer of placement control. Patient flow centers, command centers, electronic dashboards, daily bed meetings, and placement coordination teams can give staff a view of capacity beyond their own wards and help them manage cases across service boundaries [19,35,36].

That shared view is valuable, but it is still only the start of a placement decision. The receiving service must decide whether the patient fits its specialty scope, whether staffing is sufficient, whether infection-control requirements can be met, and whether the ward’s routines can absorb the case. A visible bed therefore remains only a candidate bed until these local conditions are satisfied.

Limited centralization is most plausible when the shared pool is built around clinically adjacent services. General medical units, geriatric medicine, and low-acuity overflow areas may be able to share capacity because their staffing patterns and care routines overlap. In such cases, reassignment is less likely to require a full renegotiation of clinical responsibility.

Other beds are harder to move. Specialist teams, equipment needs, isolation rules, and specialty accountability may make local control more appropriate. The practical task is to distinguish beds that can be shared routinely from beds that require additional clinical approval, and from beds where ward access should remain protected. Evidence on off-service and outlying placements is a useful warning here. Overflow beds can relieve immediate pressure, but patients placed outside their primary specialty context may face added risks in quality, length of stay, readmission, and coordination [17,37,38].

The boundary between local access and central authority may also need to change as pressure rises. In ordinary periods, wards may keep priority over protected local capacity. Once emergency department boarding, admission waits, or simultaneous ward pressure passes agreed trigger points, the central bed management team may need stronger authority to release capacity across units.

The difficult part is making those triggers work while the system is under pressure. Command center evaluations show that better visibility does not guarantee smoother flow when data quality is weak, processes are poorly integrated, or units are not engaged [19]. Bed-assignment research adds a timing problem: a bed may be marked as allocated before the intended patient is ready to move, while other ready patients continue to wait [18]. Evidence on medical outliers and off-service placement raises the same concern from the clinical side [17,39]. Limited centralization is therefore workable only when shared visibility is supported by timely identification, authorization, assignment, and a placement that the receiving ward can actually use.

### 7.5. Implementation Challenges

An empty bed shown in the central system is still only a possible placement. The receiving ward must be able to take the patient safely, and that judgment depends on nursing cover, skill mix, and local support at the time of transfer. General wards often allow more sharing because their routines overlap. Beds in intensive care, isolation, postoperative specialties, pediatrics, maternity, or psychiatry are different. Equipment, staffing, infection-control requirements, and specialty accountability make these beds less flexible. Overflow or off-service placement can relieve immediate pressure, but the evidence also points to risks in quality, length of stay, readmission, and coordination work [17,37,38].

These clinical limits need to be settled in rules before congestion makes each placement harder. Hospitals need to know which unit accepts the patient, which team carries clinical responsibility, how escalation is triggered, and when central coordinators can override local claims on capacity. If those decisions are left until the system is already under pressure, the shared pool may add delay rather than flexibility. Protocols, accountability arrangements, and the authority given to the bed management team therefore shape whether shared capacity can be used in practice [35].

Information systems have to support these rules at the moment decisions are made. Bed teams need current information on bed status, room constraints, expected discharges, transfer progress, and pending placement decisions. Dashboards and bed-management platforms can help, but command-center studies show that data quality and departmental engagement still affect performance [19,36,40]. A listed bed is not enough. Staff must be able to identify it, clear its use, and complete the placement while the demand state still matters.

## 8. Conclusions

### 8.1. Summary and Main Findings

The same stock of beds can be governed by giving wards and the central system different degrees of access. In the α framework, full decentralization, bounded centralization, and full centralization are positions on a single continuum between immediate ward access and hospital-wide coordination. When reassignment is reliable and almost frictionless, full centralization remains the benchmark because it gives the hospital the widest feasible allocation set after demand is observed.

The later results do not overturn that benchmark. They show where it begins to need qualification. Coordination friction can move the numerical maximizer away from full centralization, but this shift may have limited managerial meaning when the performance difference is small. The interior boundary becomes more important when high pressure reduces the reliability or usable share of pooled reassignment, and when capacity close to the ward can absorb pressure before delays spread. Local protection has value in this narrower sense. It is useful when it can be reached at the right time and used with sufficient reliability, not merely because it is locally held.

These findings still support centralized pooling as a strong reference point, but they change the practical test for reform. Managers need to ask how much pooled capacity the hospital can identify, authorize, staff, and convert into placements while the demand state remains active. Because the evidence comes from a stylized, mechanism-oriented simulation, the framework should be used for hospital-specific assessment rather than as a universal prescription for α.

### 8.2. Limitations and Future Research

The simulations are intended to make one governance mechanism easier to see, rather than to recommend a policy for any specific hospital. The framework has not been calibrated with hospital-level operational data, and its results have not been validated against observed placement records. This controlled setting is useful because it separates the formal scope of pooling from the capacity that can actually be used. It does not, however, produce an α value that is ready to be implemented.

Net bed demand is the main simplification that makes the model tractable. It combines admission pressure, continuing occupancy, and expected bed releases into a single variable, so the comparison across governance regimes remains manageable. That choice also leaves some important dynamics outside the main framework. Length of stay, discharge timing, and changing occupancy are not modeled directly. Future work could bring these elements into the analysis through a rolling-horizon allocation model or a Markov occupancy model.

For empirical validation, the first task would be to rebuild the net demand variable from hospital records. Relevant sources would include admission requests, current occupancy, expected and actual discharges, transfer requests, and time stamped placement decisions. Several operating constraints are also simplified in the present framework. Staffing availability, skill mix, patient acuity, specialty compatibility, infection-control requirements, clinical authorization, and transfer execution enter only through reduced form coordination and stress-state imperfection terms. Command center records, bed management platforms, transfer logs, approval timestamps, and placement histories could make these mechanisms more observable.

A hospital-level test could then follow the boundary as it is used in practice. Ward-level net demand and the realized split between locally accessible and centrally coordinated capacity would need to be reconstructed first. Coordination burden could be approximated from placement timestamps, approval delays, transfer logs, and the number of wards involved in a reassignment. High-pressure states could be identified from emergency department boarding, high occupancy, simultaneous ward shortages, or admission waiting peaks. The gap between beds visible in a central pool and beds converted into timely placements would provide a trace of stress-state usability loss. These boundary patterns could then be compared with delayed admission, off-service placement, length of stay, readmission, transfer to higher acuity care, and waiting time [17,19,40].

One further limitation concerns the scale of the boundary. The present model uses a hospital-wide boundary, while many hospitals operate with service-line clusters, specialty overflow rules, or hybrid command center structures. Extending the α framework in that direction would allow future work to distinguish routine sharing within compatible services from less routine reassignment across specialty boundaries.

## Figures and Tables

**Table 1:** Notation and model primitives for the governance framework.

Category	Symbol	Meaning
System	i∈I={1,…,n}	Ward index and ward set
Capacity	$B_{i}$	Nominal bed capacity of ward i
	$B=\sum_{i=1}^{n}B_{i}$	Total hospital bed stock
Demand	$D_{i}$	Net bed demand faced by ward i during the planning period
	$D=(D_{1},\ldots ,D_{n})$	Ward-level demand vector
	μ	Mean vector of ward-level demand
	Σ	Covariance matrix of ward-level demand
	$\rho_{ij}$	Demand correlation between wards i and j
Governance	α∈[0,1]	Degree of centralization; governance boundary between local protection and shared pooling
	$r_{i}(\alpha )=(1-\alpha )B_{i}$	Protected local reserve of ward i
	S(α)=αB	Hospital-wide shared-pool capacity
Allocation	$x_{i}(D,\alpha )$	Effective capacity assigned to ward i after demand is realized
Outcomes	$A_{i}(D,\alpha )$	Admitted demand in ward i
	$U_{i}(D,\alpha )$	Idle capacity in ward i
	$L_{i}(D,\alpha )$	Unmet demand in ward i
Costs	$p_{i}$	Unit shortage penalty for ward i
	$h_{i}$	Unit idle-capacity cost for ward i
Coordination	c	Weight on coordination friction
	Q(D,α)	Realized coordination burden under allocation x(D,α)
	$Q_{m}(D,\alpha )$	Alternative coordination-friction specification, with m=1,…,4
Tail risk	$L_{sys}(D,\alpha )=\sum_{i}L_{i}(D,\alpha )$	Total system shortage
	η	Weight placed on tail-risk exposure
	γ	Convexity parameter for severe-shortage states
Performance	$C_{avg}(\alpha )$	Expected operating loss
	$C_{tail}(\alpha )$	Tail-risk exposure
	Π(α)	Overall governance performance

**Table 2:** Interpretation of coordination-friction specifications.

Measure	What the Measure Emphasizes	Interpretation in Bed Management
$Q_{1}$	Deviation from the ward’s nominal capacity base	This is the main specification. It treats coordination work as increasing when the realized assignment moves capacity away from its usual ward base. This is useful because it charges FD, BC, and FC according to the realized reassignment, not according to the name of the regime.
$Q_{2}$	Amount of shared-pool capacity used	This specification reads coordination burden as the workload generated by activating the shared pool. It is relevant when the central bed-management team has to identify, release, and assign a larger amount of pooled capacity.
$Q_{3}$	Number of wards receiving pooled capacity	This specification focuses on the spread of coordination. The burden may increase when more wards are involved, even if the total amount reassigned is not large, because more communication, approval, and handoff decisions are needed.
$Q_{4}$	Ward-specific difficulty of reassignment	This specification allows some wards to be more difficult to reassign to or from. The weight may be higher for wards with stricter staffing, specialty fit, infection control, or clinical governance requirements.

**Table 3:** Practical interpretation of key governance parameters.

Parameter	Role in the Model	Practical Interpretation for Hospital Bed Management
α	Governance boundary between protected local reserve and hospital-wide pooling.	A higher α opens more ward capacity to central coordination; a lower α preserves more immediate ward-level access. It is a planning-level boundary, not a real-time dispatching rule.
c	Weight on realized coordination friction Q(D,α).	Captures how difficult, slow, or costly cross-ward reassignment is. The burden may come from authorization, patient-ward fit checks, transfer work, staffing limits, or receiving-ward coordination.
η	Weight placed on tail-risk exposure.	Indicates how much severe-shortage states matter beyond average operating loss. A higher η gives more importance to avoiding system-wide or clinically difficult shortages.
γ	Convexity of the severe-shortage penalty.	Determines how sharply the penalty increases when shortages become large or simultaneous across wards. A higher γ reflects disproportionate concern for escalation, ED boarding, or broader flow disruption.
δ	Stress-state pooled-usability loss.	Measures how much pooled capacity becomes less usable under high-pressure states. It represents loss of timely usability, not physical bed loss.

**Table 4:** Clinical-operational mapping of model components and observable indicators.

Model Domain	Model Component	Operational Reading in Hospital Bed Management	Possible Empirical Traces
Demand	$D_{i}$	Net bed pressure on ward i during the planning period, combining admission pressure, continuing occupancy, and expected bed releases.	Beginning occupancy, admission requests, discharge records, transfer inflows and outflows.
Governance boundary	α	Planning-level degree to which ward capacity is made available for hospital-wide coordination rather than kept under immediate ward access.	Bed-pooling policies, central allocation rules, scope of beds managed by the bed-control team.
Local access	$r_{i}(\alpha )$	Capacity that ward i can use without first entering a central reassignment process.	Protected-bed rules, service-line reserves, ward-level bed-holding practices.
Shared capacity	S(α)	Capacity visible to the central bed-management process and formally eligible for cross-ward assignment.	Bed-board entries, centrally managed bed lists, beds released to the hospital-wide pool.
Realized allocation	$x_{i}(D,\alpha )$	Final effective placement or capacity assignment to ward i after demand has been observed.	Final ward placement, off-service admissions, reassignment records.
Coordination burden	Q(D,α)	Work needed to turn pooled capacity into usable capacity across wards.	Transfer delays, approval steps, handoffs, ward acceptance decisions.
Coordination intensity	c	Reduced-form weight on the difficulty, delay, or cost of cross-ward reassignment.	Staffing gaps, administrative bottlenecks, incomplete bed-status information, weak IT support.
Stress condition	Stress states	High-pressure demand states in which several wards compete for usable capacity at the same time.	High occupancy, ED boarding, admission waiting peaks, simultaneous ward shortages.
Pooled-capacity usability	$\lambda_{s},\delta$	Loss of effective pooled-capacity usability when the system is under stress.	Unstaffed beds, specialty mismatch, delayed placement, rejected placement requests.
Tail-risk concern	η,γ	Additional concern for severe or simultaneous shortages beyond average operating loss.	Escalation events, severe boarding, multi-ward shortage episodes, urgent capacity alerts.

**Table 5:** Threshold and simulation robustness of benchmark classifications.

Robustness Check	Scenario	Test Setting	Raw $\alpha^{*}$	$\Delta_{BC-FC}$	Classification	Interpretation
Threshold sensitivity	Frictionless benchmark	$\epsilon_{\Delta }=0.25,0.5,1.0$	1.00	0.0000	FC-consistent for all thresholds	FC remains on the performance plateau.
Threshold sensitivity	Costly pooling only	$\epsilon_{\Delta }=0.25,0.5,1.0$	0.26	0.0453	FC-comparable for all thresholds	The raw interior gain is too small to be substantive.
Threshold sensitivity	Costly pooling + mild stress imperfection	$\epsilon_{\Delta }=0.25,0.5,1.0$	0.20	4.7138	BC for all thresholds	The interior boundary remains substantively better than FC.
Monte Carlo stability	Frictionless benchmark	N=20,000	1.00	0.0000	FC-consistent	The frictionless benchmark is unchanged.
Monte Carlo stability	Costly pooling only	N=20,000	0.26	0.0366	FC-comparable	The small interior gain remains practically negligible.
Monte Carlo stability	Mild stress-imperfection baseline	N=20,000	0.20	4.7190	BC	The substantive interior pattern is stable.

$\Delta_{BC-FC}=\underset{\alpha \in (0,1)}{max}\;\hat{\Pi }(\alpha )-\hat{\Pi }(1)$. The threshold $\epsilon_{\Delta }$ is used only for substantive classification; it does not affect simulated performance values or the raw maximizing α. The Monte Carlo stability checks use N=20,000 demand scenarios.

**Table 6:** Multi-ward extension under fully effective and stress-imperfect pooling.

System Size	Pooling Condition	Raw $\alpha^{*}$	$\Delta_{BC-FC}$	Classification	Interpretation
n=2	No imperfection	0.26	0.0453	FC-comparable	The raw optimizer is interior, but the gain over FC is practically small.
n=2	Mild stress imperfection	0.20	4.7138	BC	The canonical case shows a substantive interior boundary.
n=5	No imperfection	0.46	0.0236	FC-comparable	The raw interior gain remains practically negligible.
n=5	Mild stress imperfection	0.24	12.5639	BC	The interior-boundary pattern appears in a larger ward system.
n=10	No imperfection	0.70	0.0097	FC-comparable	FC remains performance-comparable despite an interior raw maximizer.
n=10	Mild stress imperfection	0.26	29.8877	BC	The interior-boundary pattern persists in the larger system.

$\Delta_{BC-FC}$ is defined in (34). “No imperfection” refers to fully effective pooled allocation. “Mild stress imperfection” uses δ=0.02. Substantive classification follows (35) with $\epsilon_{\Delta }=0.5$. Larger $\Delta_{BC-FC}$ values in larger systems partly reflect aggregate system-level cost accounting rather than calibrated hospital-level policy gains.

**Table 7:** Summary of computational scenarios and robustness analyses.

Evidence Covered	Analysis/Source	Key Finding	Interpretation
Benchmark scenarios	Figure 4a–c	FC is performance-consistent in the frictionless case; costly pooling alone is FC-comparable; mild stress imperfection yields substantive BC.	The main shift toward BC occurs only when pooled capacity remains useful but becomes less reliable under pressure.
Mechanism and boundary	Figure 5; Figure 6 (c-δ grid)	The interior pattern reflects several cost components and appears only in part of the c-δ space.	BC is conditional on operating regions with coordination burden and stress-state usability loss.
Threshold and simulation robustness	Table 5	Classifications are preserved when $\varepsilon_{\Delta }$ is varied and when N is increased.	The FC-comparable versus BC distinction is not driven by one cutoff or one simulation sample.
Alternative friction specifications	Appendix Table A2	The stress-imperfection case remains BC under $Q_{1}$–$Q_{4}$ specifications.	The mechanism is robust to alternative reduced-form measures of coordination burden.
Multi-ward extension	Table 6; Appendix Figure A1	For larger n, fully effective pooling remains FC-comparable, while stress imperfection restores BC.	The mechanism is not confined to the two-ward case, though results remain mechanism checks rather than calibrated hospital-size comparisons.

$\Delta_{BC-FC}$ is defined in Equation (34), and $\varepsilon_{\Delta }$ denotes the substantive-improvement threshold. Robustness refers to the qualitative classification and mechanism pattern, not to identical numerical values of $\alpha^{*}$ or $\Delta_{BC-FC}$ across all settings.

## Data Availability

The original contributions presented in this study are included in the article. The computational analysis is based on simulated scenarios and parameter settings described in the manuscript. No patient-level, identifiable human, clinical-record, survey, or third-party data were used. Further inquiries can be directed to the corresponding author.

## Appendix A. Additional Computational Details

This appendix provides additional details on the computational experiments, including the parameter settings, allocation procedure, robustness checks, and multi-ward extensions. Table A1 reports the baseline values used in the main experiments and the robustness dimensions along which those values are varied. The experiments are not calibrated to a specific hospital. The baseline values define the reference high-pressure setting studied in the main text, while the robustness checks examine whether the qualitative classifications change when key inputs such as demand correlation, coordination friction, pooled-allocation imperfection, improvement thresholds, and simulation size are varied.

**Table A1:** Baseline experimental parameter settings and robustness dimensions.

Category	Parameter	Baseline Setting	Robustness/Sensitivity
System size	n	2	5,10
Capacity	B	(50,50)	Heterogeneous $B_{i}$ in multi-ward tests
Demand pressure	$\kappa_{i}=\mu_{i}/B_{i}$	(0.96,0.92)	Varied in multi-ward settings
Demand mean	μ	(48,46)	Implied by $\kappa_{i}B_{i}$
Demand variability	cv	0.17	$cv_{i}\in [0.12,0.20]$ in multi-ward tests
Demand correlation	ρ	0.4	0 to 0.9
Idle cost	$h_{i}$	1	Fixed baseline
Shortage cost	$p_{i}$	(10,12)	Heterogeneous penalties in multi-ward tests
Coordination friction	c	2.0	0 to 10
Tail-risk weight	η	0.05	Fixed in baseline checks
Tail convexity	γ	2	Fixed in baseline checks
Pooled imperfection intensity	δ	0.02	0 to 0.08; refined low-δ grid
Effectiveness lower bound	$\lambda_{min}$	0.94	Fixed
Stress-state definition	—	Upper 15% of total demand	Fixed
Governance grid	α	0,0.02,…,1	Fixed
Baseline coordination measure	Q	$Q_{1}$	$Q_{2},Q_{3},Q_{4}$
Practical improvement threshold	$\epsilon_{\Delta }$	0.5	0.25,1.0
Monte Carlo sample size	N	12,000	20,000 for stability checks

The baseline coordination measure is $Q_{1}$, defined in (10). Alternative definitions $Q_{2},Q_{3},Q_{4}$ are normalized by their reference expected value under α=1 and c=0, so that different friction specifications can be compared on a common scale. The practical threshold $\epsilon_{\Delta }$ is used only for reporting substantive regime classifications; it does not affect the performance calculation or the choice of the raw maximizing α.

### Appendix A.1. Allocation Procedure

Algorithm A1 describes the scenario-level allocation and performance-evaluation procedure used in the computational experiments. The procedure is applied for each candidate governance boundary α∈A, where A={0,0.02,…,1}. The algorithm operationalizes the definitions in Section 4 and Section 6 and computes local reserves, shared-pool capacity, stress-state pooled effectiveness, realized allocations, coordination burden, cost components, and the estimated performance value $\hat{\Pi }(\alpha )$.

**Algorithm A1. Scenario-level shared-pool allocation and performance****-****evaluation****procedure.**
**Input:** Ward capacities $B_{i}$; demand scenarios $D_{s}=(D_{s1},\ldots ,D_{sn})$, s=1,…,N; centralization grid A; shortage costs $p_{i}$; idle-capacity costs $h_{i}$; coordination-friction weight c; tail-risk weight η; tail convexity γ; demand correlation ρ; pooled-imperfection parameters $\left(\delta ,\lambda_{min}\right)$; stress-state quantile τ; coordination-friction measure $Q_{m}$; and normalization scale $q_{m}^{scale}$. **Output:** Estimated governance performance $\hat{\Pi }(\alpha )$ and average cost components for each α∈A. For each α∈A: 1. Compute protected local reserve and shared-pool capacity: $$r_{i}(\alpha )=(1-\alpha )B_{i},$$ $$S(\alpha )=\alpha \sum_{i=1}^{n}B_{i}.$$ 2. Identify stress-state scenarios. Let $$T_{s}=\sum_{i=1}^{n}D_{si}.$$ Scenario s is classified as a stress-state if $T_{s}$ is no smaller than the τ-quantile of $\{T_{s}{\}}_{s=1}^{N}$. Denote the set of stress-state scenarios by $S^{stress}$. 3. Compute the pooled-allocation effectiveness factor for each scenario: $$\lambda_{s}=\left\{\begin{array}{ll} 1, & \mathrm{if}\;\mathrm{pooled}\;\mathrm{imperfection}\;\mathrm{is}\;\mathrm{disabled}\;\mathrm{or}\;\delta =0,\\ max\{\lambda_{min},\;1-\alpha \delta (1+\rho )1_{s\in S^{stress}}\}, & \mathrm{otherwise}. \end{array}\right.$$ 4. Compute the remaining service need after protected local capacity: $$R_{si}=max\{D_{si}-r_{i}(\alpha ),0\}.$$ Convert this to the physical shared-pool requirement: $${\tilde{R}}_{si}=\frac{R_{si}}{\lambda_{s}}.$$ 5. Initialize shared-pool allocation and remaining shared-pool capacity: $$y_{si}=0,\;\;P_{s}=S(\alpha ).$$ 6. Compute ward-level allocation scores. Let $$\bar{\lambda }=\frac{1}{N}\sum_{s=1}^{N}\lambda_{s}.$$ For each ward i, define $$a_{i}=\bar{\lambda }p_{i}-\frac{c\;\xi_{i}}{q_{m}^{scale}},$$ where $$\xi_{i}=\left\{\begin{array}{ll} \omega_{i}, & \mathrm{if}\;Q_{m}=Q_{4},\\ 1, & \mathrm{otherwise}. \end{array}\right.$$ Order wards by decreasing $a_{i}$, and retain only wards with $a_{i}>0$. 7. Allocate the shared pool greedily according to this order. For each retained ward i, $$y_{si}=min\{{\tilde{R}}_{si},P_{s}\},$$ $$P_{s}=P_{s}-y_{si}.$$ 8. Compute physical and effective allocations: $$x_{si}^{phys}=r_{i}(\alpha )+y_{si},$$ $$x_{si}^{eff}=r_{i}(\alpha )+\lambda_{s}y_{si}.$$ 9. Compute ward-level outcomes: $$L_{si}=max\{D_{si}-x_{si}^{eff},0\},$$ $$U_{si}=max\{x_{si}^{eff}-D_{si},0\}.$$ Here $L_{si}$ denotes unmet demand and $U_{si}$ denotes idle ward capacity. The unused shared-pool capacity in scenario s is $P_{s}$. 10. Compute the coordination-friction measure. The baseline specification is $$Q_{1}(D_{s},\alpha )=\frac{1}{2}\sum_{i=1}^{n}|x_{si}^{phys}-B_{i}|.$$ The alternative specifications used in robustness checks are $$Q_{2}(D_{s},\alpha )=\sum_{i=1}^{n}y_{si},$$ $$Q_{3}(D_{s},\alpha )=\sum_{i=1}^{n}1\{y_{si}>0\},$$ $$Q_{4}(D_{s},\alpha )=\frac{1}{2}\sum_{i=1}^{n}\omega_{i}|x_{si}^{phys}-B_{i}|.$$ The normalized coordination burden is $$Q_{s}^{norm}=\frac{Q_{m}(D_{s},\alpha )}{q_{m}^{scale}}.$$ 11. Compute scenario-level cost components: $$C_{s}^{short}=\sum_{i=1}^{n}p_{i}L_{si},$$ $$C_{s}^{idle}=\sum_{i=1}^{n}h_{i}U_{si}+\bar{h}P_{s},\;\;\bar{h}=\frac{1}{n}\sum_{i=1}^{n}h_{i},$$ $$C_{s}^{coord}=cQ_{s}^{norm},$$ $$L_{s}^{sys}=\sum_{i=1}^{n}L_{si},$$ $$C_{s}^{tail}=\eta (L_{s}^{sys}{)}^{\gamma }.$$ 12. Compute total scenario loss: $$C_{s}^{total}=C_{s}^{short}+C_{s}^{idle}+C_{s}^{coord}+C_{s}^{tail}.$$ 13. Estimate performance under α: $$\hat{\Pi }(\alpha )=-\frac{1}{N}\sum_{s=1}^{N}C_{s}^{total}.$$ Also record average shortage cost, idle-capacity cost, coordination-friction cost, tail-risk cost, normalized coordination burden, unused shared-pool capacity, and average pooled-allocation effectiveness. End for. Return $\hat{\Pi }(\alpha )$ and all recorded cost components for each α∈A.

After Algorithm A1 has been applied over the full grid A, the raw maximizing boundary is obtained as $arg\underset{\alpha \in A}{max}\;\hat{\Pi }(\alpha )$. The improvement of the best bounded-centralization design over FC is computed as $\Delta_{BC-FC}=\underset{\alpha \in (0,1)}{max}\;\hat{\Pi }(\alpha )-\hat{\Pi }(1)$. The substantive classification then follows the threshold rule in Equation (35). Thus, the threshold affects only the reporting classification; it does not affect the estimated performance values or the raw maximizing value of α.

### Appendix A.2. Additional Robustness Results

Table A2 reports the same stress-state imperfection experiment under alternative normalized coordination-friction measures. The baseline measure $Q_{1}$ follows the reallocation-intensity interpretation used in the main text. The alternatives capture different aspects of the same coordination problem: $Q_{2}$ emphasizes the amount of shared-pool capacity used, $Q_{3}$ emphasizes the number of wards brought into pooled allocation, and $Q_{4}$ allows reassignment difficulty to vary across wards. This check examines whether the interior-boundary pattern is tied to treating coordination burden only as deviation from nominal ward capacity.

**Table A2:** Robustness to alternative coordination-friction measures.

Measure	Definition	Raw $\alpha^{*}$	$\Delta_{BC-FC}$	Classification
$Q_{1}$	$Q_{1}=\frac{1}{2}\sum_{i}|x_{i}-B_{i}|$	0.20	4.7138	BC
$Q_{2}$	$Q_{2}=\sum_{i}y_{i}$	0.22	6.1097	BC
$Q_{3}$	$Q_{3}=\sum_{i}1\{y_{i}>0\}$	0.20	4.7864	BC
$Q_{4}$	$Q_{4}=\frac{1}{2}\sum_{i}\omega_{i}|x_{i}-B_{i}|$	0.20	4.7160	BC

The definitions of $Q_{1}$–$Q_{4}$ follow (10)–(13). In Table A2, $y_{i}$ denotes the realized shared-pool allocation to ward *i*, consistent with Algorithm A1. $\Delta_{BC-FC}$ is defined in Equation (34). All Q measures are normalized by their reference expected value under α=1 and c=0, so the reported comparisons are on a common scale. The table reports the baseline stress-imperfection setting. These checks do not establish robustness under every possible coordination-cost specification; they show that the interior-boundary pattern is not eliminated by the alternative normalized Q definitions considered here.

### Appendix A.3. Additional Multi-Ward Results

Figure A1 reports the full α-grid performance curves for the multi-ward extensions. These curves supplement the summary statistics reported in the main text. They are included to assess whether the qualitative pattern observed in the two-ward canonical case persists when the number of wards increases.
